# B-type Plexins promote the GTPase activity of Ran to affect androgen receptor nuclear translocation in prostate cancer

**DOI:** 10.1038/s41417-023-00655-6

**Published:** 2023-08-10

**Authors:** Ritu Garg, Sofia Endzhievskaya, Magali Williamson

**Affiliations:** 1 School of Cancer and Pharmaceutical Sciences, Faculty of Life Sciences & Medicine, King’s College London, London, UK; 2 Randall Division of Cell and Molecular Biophysics, Faculty of Life Sciences & Medicine, King’s College London, London, UK

**Keywords:** Prostate cancer, Cell biology, RNAi

## Abstract

Resistance to anti-androgen therapy for metastatic prostate cancer is a major clinical problem. Sema3C promotes resistance to androgen withdrawal via its receptor, PlexinB1. Activation of PlexinB1 promotes the ligand-independent nuclear translocation of the androgen receptor (AR), which may contribute to resistance to androgen deprivation therapy. However, the mechanism by which PlexinB1 promotes nuclear translocation is unclear. We show here that PlexinB1 and B2 regulate nuclear import by acting as GTPase activating proteins (GAPs) for the small RasGTPase Ran, a key regulator of nuclear trafficking. Purified PlexinB1/B2 protein catalyses the hydrolysis of RanGTP, and mutations in the GAP domain of PlexinB1 inhibit this activity. Activation of PlexinB1/B2 with Sema4D decreases the levels of RanGTP, while PlexinB1 or B2 depletion increases the levels of activated Ran in the cell. Ran directly associates with B-type plexins in a GTP-dependent manner. Sema4D is internalised by endocytosis, and PlexinB1 and Ran display overlapping patterns of expression. Furthermore, Sema4D/PlexinB1-induced AR nuclear translocation is dependent on the GAP domain of PlexinB1 and is blocked by the expression of non-functional Ran mutants. Depletion of PlexinB1 decreases the nuclear/cytoplasmic ratio of Ran, indicative of a higher RanGTP/GDP ratio. Plexins may promote the growth of androgen-independent prostate cancer through their activity as RanGAPs.

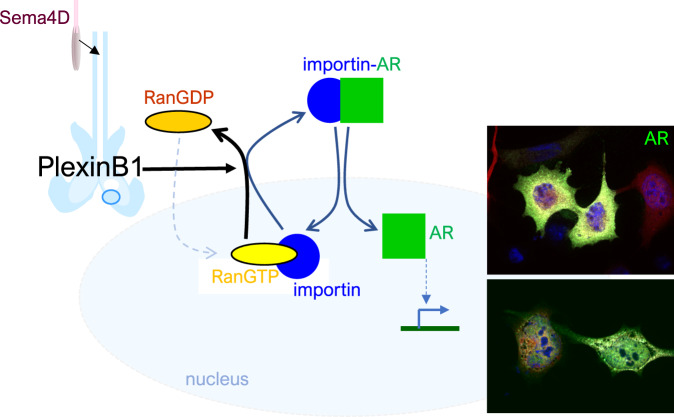

## Introduction

The growth of primary prostate cancer is dependent on the androgen receptor (AR) and AR signalling is critical to prostate cancer progression [1]. Androgen deprivation therapy (ADT), the first line of treatment for progressive prostate cancer, is effective at first but eventually fails due to the development of resistance, primarily through androgen-independent reactivation of AR [2]. Upregulation of the glucocorticoid receptor (GR) [3], as well as ligand-independent AR activation, promote ADT resistance [1]. AR and GR activity is dependent on the translocation of both receptors to the nucleus, where they act as transcription factors [4].

Translocation of most proteins, including ligand-bound AR [5] and GR [6], in and out of the nucleus is controlled by the small GTPase, Ras-related nuclear protein (Ran). Ran cycles between a GTP-bound active form and a GDP-bound inactive form [7]. RanGDP is converted to RanGTP by its chromatin-bound guanine exchange factor (GEF), Regulator of chromosome condensation 1 (RCC1) [8], while RanGAP1, present in the cytoplasm, catalyses the intrinsic GTPase activity of Ran, converting RanGTP to RanGDP [9, 10]. The spatial separation of these regulators of Ran creates a RanGTP gradient [11] across the nuclear envelope in interphase cells which confers positioning cues. RanGTP is thought to function by inducing the localised release of regulatory factors from inhibitory complexes made between karyopherins (exportins and importins) [12] and cargo proteins containing nuclear localisation sequences (NLS) or nuclear export sequences (NES).

During nuclear import, importins bind to NLS-containing cargo proteins in the cytosol and traffic them into the nucleus through the nuclear pore complex [13, 14]. Cargo proteins are liberated from importin-α/β -cargo complexes by the binding of RanGTP to the importin in the nucleus. The RanGTP-importin complex then exits the nucleus and dissociates in the cytoplasm upon hydrolysis of RanGTP to RanGDP, catalysed by RanGAP1 in the cytoplasm [9, 15]. During nuclear export, a complex of RanGTP, exportin (CRM1) and NES-containing cargo exit the nucleus via the nuclear pore. Dissociation of the RanGTP-CRM1-cargo complex and release of export cargo into the cytoplasm ensues upon conversion of the RanGTP to RanGDP, catalysed by RanGAP1 [13]. RanGDP is recycled back to the nucleus upon binding to nuclear transport factor 2 (NTF2) [16]. Deregulation of Ran in cancer has been reported in several tissue types [17].

Plexins are transmembrane receptors for semaphorins, a group of membrane-bound or secreted extracellular cell-guidance molecules [18]. Vertebrates possess nine plexin genes, classified into 4 classes (class A(1–4), B(1–3) C1 and D1) [19]. PlexinB1 and PlexinB2 are the receptors for semaphorin 4D (Sema4D) [20]. B-class plexins regulate several small GTPases, interacting with Rac [21], Rnd1-2-3 [22], R-Ras [23], M-Ras [24] and RhoD [25] and regulating Rho via PDZRhoGEF/LARG [26] and p190RhoGAP [27]. The cytoplasmic part of plexins contains two regions of homology to RasGAPs, which fold together to form a GTPase activating protein (GAP) domain [28], and plexins act directly as GAPs for Rap1B, Rap2A [29]. B-class plexins also activate and are activated by the receptor tyrosine kinases ErbB2 [30] and Met [31] and regulate axon pathfinding, cell morphology and cell motility [19] and have been implicated in many cancers, including prostate cancer [32]. PlexinB1/B2 activation promotes nuclear trafficking of both AR [33] and GR [34] as well as the NF-kB subunit, p65 [35] (Supplementary Fig. 1) and YAP [36]. The mechanism by which plexins regulate the nuclear trafficking of AR, GR and other transcription factors is not known.

Sema3C signalling via PlexinB1 promotes castration-resistant prostate tumour growth in mouse models [32]. Furthermore, Sema3C inhibition delays castrate-resistant prostate cancer (CRPC) and enzalutamide-resistant progression [32]. Resistance to androgen deprivation therapy arises from ligand-independent translocation of AR and/or GR to the nucleus, and Plexin activation promotes this process [2–4]. Here we investigate the mechanism by which plexin signalling promotes nuclear translocation.

## Results

### Sema4D-induced translocation of AR to the nucleus is dependent on Ran signalling

Activation of PlexinB1 increases the translocation of AR [33], GR [34] and RelA [35] (Supplementary Fig. 1) to the nucleus, and nuclear trafficking of most proteins is controlled by the small GTPase, Ran [7]. Furthermore, Sema4D/PlexinB1-driven GR nuclear translocation requires an intact NLS1 in GR [34], implicating the importin/Ran system in the process. These findings led us to question whether PlexinB1 may exert its role in nuclear trafficking by affecting RanGTPase activity.

**Fig. 1:**
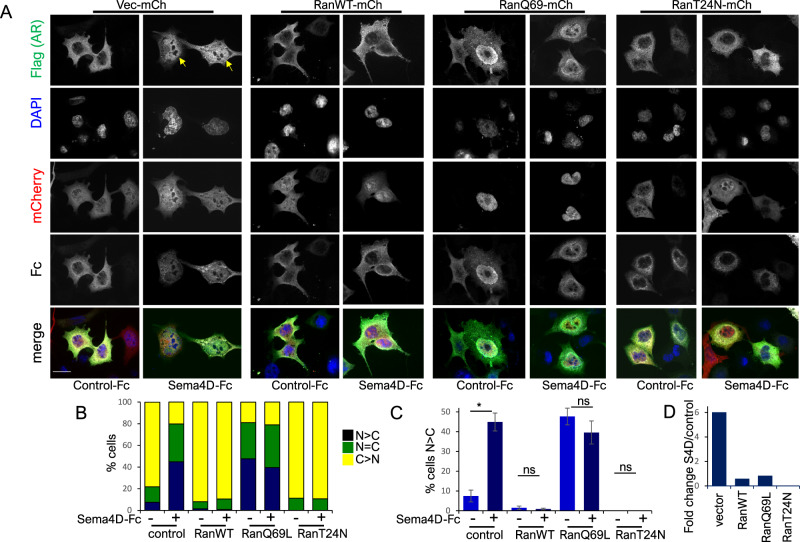
Sema4D-stimulated translocation of AR to the nucleus is dependent on Ran signalling. **A** Representative images of serum-starved PC3 cells transfected with AR-Flag variant 2 (green), and pmCherry, pmCherry-RanWT, pmCherry-RanQ69L, or pmCherry-RanT24N (red), together with Fc empty vector or Sema4D-Fc (grey). Arrows denote nuclear AR (×60 magnification scale bar = 25 μm). **B** % cells in which intensity of immunostaining for nuclear AR was greater (N > C), equal (N = C), or less (N < C) than cytoplasmic staining in PC3 cells co-transfected with AR-Flag variant 2, and pmCherry, pmCherry-RanWT, pmCherry-RanQ69L, or pmCherry-RanT24N, together with Fc empty vector or Sema4D-Fc. **C** Relative number of cells in which staining of AR in the nucleus exceeded that in the cytoplasm following co-transfection with AR-Flag variant 2, and pmCherry, pmCherry-RanWT, pmCherry-RanQ69L, or pmCherry-RanT24N, together with Fc empty vector or Sema4D-Fc. *n* ≥ 50 per condition per three independent experiments (*p = <0.05, two-tailed Student’s *t*-test, ns, not significant). **D** Fold change in nuclear AR in Sema4-Fc transfected/control cells, in cells transfected with AR-flag and pmCherry, pmCherry-RanWT, pmCherry-RanQ69L, or pmCherry-RanT24N.

To investigate whether Sema4D-induced AR trafficking to the nucleus is dependent on Ran, we tested the effect of blocking Ran signalling on Sema4D-stimulated import of AR-Flag, by co-expression of non-functional mutant forms of Ran. Prostate cancer cells PC3, which lack endogenous AR, were transfected with AR-Flag together with Sema4D-Fc or vector-Fc, with or without WT or non-functional mutant forms of Ran—RanQ69L or T24N. The Q69L mutant form of Ran has strongly reduced GTPase activity, while the T24N mutant form has a low binding affinity for GTP and GDP. Transfection with AR-Flag and Sema4D-Fc and empty mCherry vector resulted in an increase in the number of cells where nuclear AR exceeded cytoplasmic AR in comparison with cells transfected with AR-Flag and control Fc-vector. Co-transfection of AR-Flag with RanWT-mCherry, RanQ69L-mCherry or RanT24N-mCherry blocked the increase in nuclear AR induced by Sema4D-Fc transfection (Fig. 1A–D) as well as DHT-induced AR translocation (Supplementary Fig. 2). Consistent with these results, expression of RanQ69L-mCherry in PC3 cells blocked the increase in nuclear AR observed upon treatment with purified Sema4D-Fc protein added to the medium (Supplementary Fig. 3). Co-transfection of AR-flag with RanWT-mCherry, RanQ69L-mCherry or RanT24N-mCherry also blocked Sema4D-induced AR nuclear translocation in MCF7 cells (Supplementary Fig. 4).

These results suggest that Sema4D-induced translocation of AR is dependent on RanGTPase signalling.

### Sema4D-induced translocation of AR to the nucleus is dependent on the GAP domain of PlexinB1

**Fig. 2:**
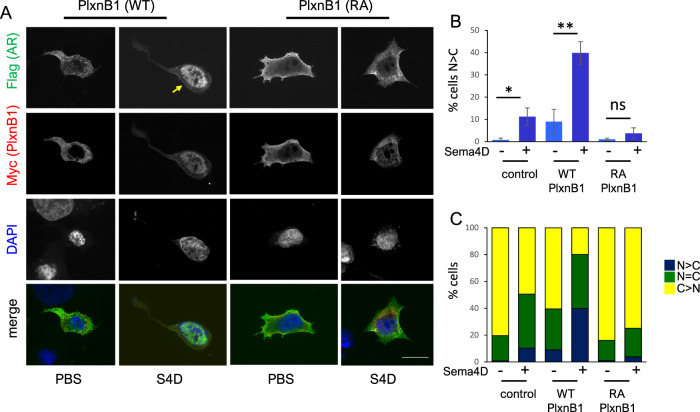
Sema4D-stimulated translocation of AR to the nucleus is dependent on the GAP domain of PlexinB1. **A** Representative images of serum-starved PC3 cells transfected with AR-Flag variant 2 (green) and PlexinB1 (WT)-myc or PlxnB1 (RA)-myc (red) and treated with vehicle (PBS) or Sema4D for 1.5 h. Arrow denotes nuclear AR (×60 magnification scale bar = 25 μm). **B** Relative number of cells in which staining of AR in the nucleus exceeded that in the cytoplasm following co-transfection with AR-Flag variant 2, and empty-vector-myc or PlexinB1 (WT)-myc or PlxnB1 (RA)-myc and treated with vehicle (PBS) or Sema4D for 1.5 h (*p = <0.05, **p = <0.01, two-tailed Student’s *t*-test, ns, not significant). **C** % cells in which intensity of immunostaining for nuclear AR was greater (N > C), equal (N = C), or less (N < C) than cytoplasmic staining in PC3 cells co-transfected with AR-Flag variant 2, and empty-vector-myc or PlexinB1 (WT) or PlxnB1 (RA) and treated with vehicle (PBS) or Sema4D for 1.5 h.

To determine whether the GAP domain of PlexinB1 is required for Sema4D-induced nuclear trafficking, PC3 cells were transfected with AR-flag and WT PlexinB1 or a mutant form of PlexinB1 (PlexinB1-RA), which lacks GAP activity [23]. Transfection of WT PlexinB1 significantly increased the level of AR in the nucleus. Additionally, stimulation of PlexinB1(WT)-transfected cells with Sema4D further increased nuclear AR (Fig. 2). In contrast, expression of the RA mutant of PlexinB1 did not increase nuclear AR with or without Sema4D treatment (Fig. 2). These results show that Sema4D-induced AR translocation to the nucleus is dependent on the GAP activity of PlexinB1.

### PlexinB1 signals via Ran to induce morphological collapse

To further establish whether Ran is an effector of PlexinB1, we examined the impact of disrupting Ran signalling on a well-established test of PlexinB1 function—the morphological collapse of Cos7 cells [37]. The collapse of Cos7 cells occurs upon stimulation of plexins with semaphorins or ectopic expression of plexins [22].

Overexpression of full-length PlexinB1 in Cos7 cells resulted in cell collapse as expected (Supplementary Fig. 5). Co-transfection of wild-type (WT) Ran with PlexinB1 also results in Cos7 cell collapse to a similar extent to transfection of PlexinB1 alone. In contrast, co-transfection of PlexinB1 with the Q69L or T24N mutant forms of Ran blocked the increase in Cos7 cell collapse resulting from PlexinB1 expression (Supplementary Fig. 5).

These results demonstrate that expression of non-functional Ran mutants blocks PlexinB1-induced collapse, suggesting that PlexinB1 signals via Ran to induce this phenotype.

### PlexinB1 depletion decreases the ratio of nuclear to cytoplasmic Ran

**Fig. 3:**
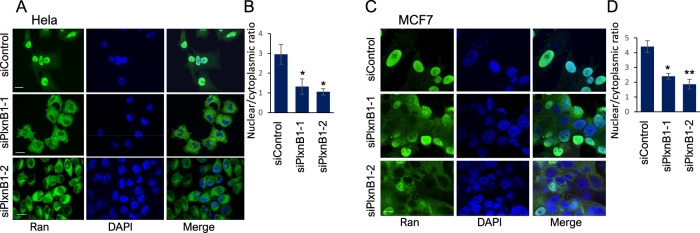
Depletion of PlexinB1 promotes the translocation of Ran to the cytoplasm. **A** HeLa cells transfected with non-silencing siRNA or siRNA to PlexinB1, stained for endogenous Ran (representative images) (scale bar = 20 μm), **B** Bar chart showing ratio of nuclear/cytoplasmic Ran staining (*n* = 4) (*p = <0.05, two-tailed Student’s *t*-test). **C** MCF7 cells transfected with non-silencing siRNA or siRNA to PlexinB1, stained for endogenous Ran (representative images) scale bar = 10 μm, **D** Bar chart showing ratio of nuclear/cytoplasmic Ran staining (*n* = 3) (*p = <0.01, **p = <0.005, two-tailed Student’s *t*-test).

During nucleocytoplasmic transport, RanGTP is hydrolysed to RanGDP in the cytoplasm, catalysed by RanGAP1 [9, 15]. RanGDP is then shuttled back to the nucleus by NTF2, which binds RanGDP specifically [16]. Consequently, failure to convert RanGTP to RanGDP, for example, due to RanGAP depletion, is predicted to result in an accumulation of RanGTP in the cytoplasm and a shift in the ratio of nuclear to cytoplasmic total Ran protein [38]. A decrease in the nuclear/cytoplasmic ratio of Ran is therefore indicative of a block in the conversion of RanGTP to RanGDP. To examine whether PlexinB1 affects the nuclear/cytoplasmic ratio of Ran, PlexinB1 expression was knocked down in HeLa, MCF and PC3 cells, and the expression levels of Ran in the nucleus and cytoplasm were recorded using immunofluorescence. Depletion of PlexinB1 resulted in a significant decrease in the nuclear/cytoplasmic ratio of total Ran protein in all three cell lines (Fig. 3, Supplementary Fig. 6), consistent with a decrease in the hydrolysis of RanGTP and a reduction in RanGAP activity.

### PlexinB1 and PlexinB2 regulate cellular levels of RanGTP

**Fig. 4:**
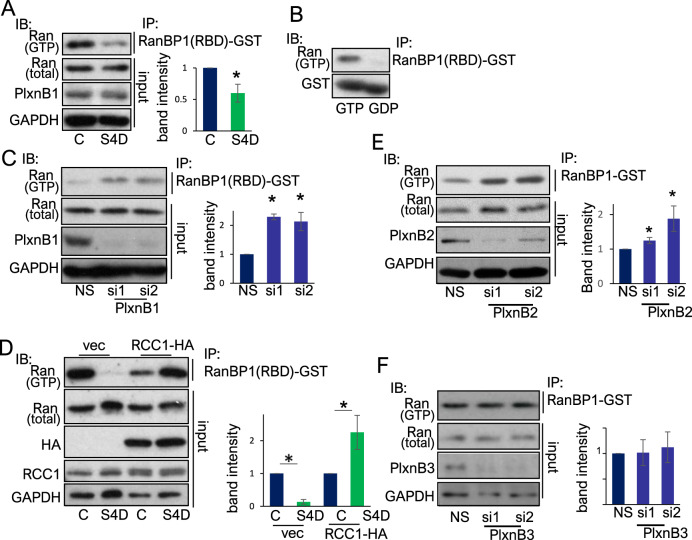
PlexinB1 and PlexinB2 regulate levels of GTP-bound Ran. **A** Stimulation of cells with Sema4D decreases the ratio of RanGTP to total Ran. Lysates of MCF7 cells treated with Sema4D or control conditioned medium for 15 min were immunoprecipitated with RanBP1(RBD)-GST (which binds to GTP-bound Ran specifically) and the ratio of RanGTP to total Ran assessed by immunoblotting. Bar chart of average band intensities (RanGTP to total Ran ratio) (*n* = 3) (*p = <0.05, two-tailed Student’s *t*-test). **B** Control for Ran activation assays; cell lysates were pre-loaded with a non-hydrolyzable form of GTP (GTPγS) or GDP, immunoprecipitated with RanBP1(RBD)-GST and immunoblotted with Ran antibody. **C** Depletion of PlexinB1 increases levels of RanGTP. Lysates of MCF7 cells, transfected with non-silencing siRNA (NS) or two different siRNAs to PlexinB1, were immunoprecipitated with RanBP1(RBD)-GST and the ratio of RanGTP to total Ran assessed by immunoblotting. Bar chart of average band intensities, RanGTP/total Ran (*n* = 3) (*p = <0.05, one-tailed Student’s *t*-test). **D** RCC1 expression reverses the Sema4D-induced reduction in RanGTP levels. MCF7 cells transfected with RCC1-HA or vector control were treated with Sema4D or control conditioned medium for 15 min and lysates immunoprecipitated with RanBP1(RBD)-GST. The ratio of RanGTP to total Ran was assessed by immunoblotting. Bar chart of average band intensities, RanGTP/total Ran (*n* = 3) (*p = <0.05, two-tailed Student’s *t*-test). **E** Depletion of PlexinB2 increases levels of RanGTP. LNCaP cells, transfected with non-silencing siRNA or two different siRNAs to PlexinB2, were lysed and immunoprecipitated with RanBP1-GST, and the ratio of RanGTP to total Ran was assessed by immunoblotting. Bar chart shows average band intensities, RanGTP/total Ran (*n* = 4) (*p = <0.05, one-tailed Student’s *t*-test). **F** Depletion of PlexinB3 does not increase levels of RanGTP. Lysates of LNCaP cells transfected with non-silencing siRNA or two different siRNAs to PlexinB3 were immunoprecipitated with RanBP1-GST, and the ratio of RanGTP to total Ran was assessed. Bar chart of average band intensities, RanGTP/total Ran (*n* = 4).

To examine whether activation of PlexinB1 has any effect on Ran activity, the levels of activated Ran (RanGTP) were measured following treatment of cells with Sema4D, a PlexinB1 agonist. MCF7, a breast cancer cell line which expresses high levels of PlexinB1, were treated with Sema4D, and RanGTP levels were detected with RanBP1(RBD)-GST, which binds to GTP-bound Ran specifically [39]. Activation of PlexinB1 with Sema4D decreased the ratio of GTP-bound Ran to total Ran (Fig. 4A). The specificity of RanBP1(RBD)-GST for RanGTP is shown in Fig. 4B. A similar decrease in RanGTP levels was also found in the prostate cancer cell line, LNCaP, upon Sema4D treatment (Supplementary Fig. 7A). Consistent with these results, depletion of PlexinB1 with siRNA resulted in an increase in GTP-bound Ran in MCF7 (Fig. 4C) and LNCaP cells (Supplementary Fig. 7B). Expression of RCC1 (a GEF for Ran) reversed the reduction in RanGTP/total Ran ratio induced by Sema4D treatment in MCF7 and LNCaP cells (Fig. 4D, Supplementary Fig. 7C). Knockdown of PlexinB2 (Fig. 4E), but not PlexinB3 (Fig. 4F), also resulted in an increase in RanGTP levels. These findings indicate that PlexinB1 and PlexinB2 have a role in the inactivation of RanGTP.

### PlexinB1 and PlexinB2 have GTPase-activating activity for Ran

**Fig. 5:**
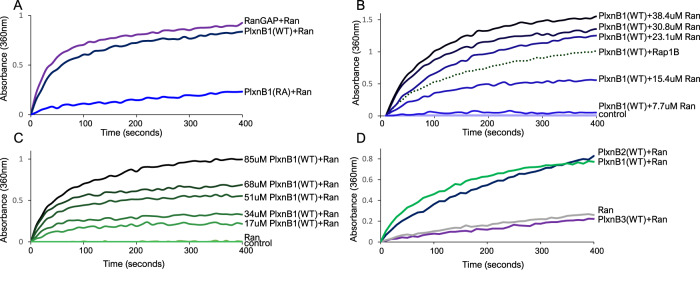
PlexinB1 and PlexinB2 act as GTPase activating proteins for Ran. **A** GTPase activating proteins (GAP) activity assays. The release of inorganic phosphate from RanGTP in the presence of 34 μM of purified cytoPlexinB1(WT), RanGAP1 or an inactive mutant form of PlexinB1 (cytoPlexinB1(RA)), which lacks GTPase catalytic activity, was measured by absorbance at 360 nm using a photometric assay over time. **B** Dependence of the RanGAP activity of PlexinB1 on Ran protein concentration. Inorganic phosphate released from RanGTP in the presence of 17.7 μM cytoPlexinB1(WT) with the various concentrations of purified RanGTP indicated. Values in the absence of Ran(control) were subtracted for each time point to control for spontaneous MESG decay and endogenous RanGTPase activity. GAP activity assay of purified cytoPlexinB1(WT) using Rap1B as substrate shown as a control. **C** Dependence of the RanGAP activity on PlexinB1 protein concentration, using 14.6 μM RanGTP and indicated PlexinB1 concentrations. Values in the absence of Ran and Plexin were subtracted for each time point to control for a slow increase in absorbance at 360 nm due to the spontaneous conversion of MESG. **D** RanGAP activity assays in the presence of 51 μM purified PlexinB1, PlexinB2 and PlexinB3 proteins or buffer control and 7.6 μM RanGTP.

Activation of PlexinB1 decreases the level of GTP-bound Ran in MCF7 and LNCaP cells (Fig. 4, Supplementary Fig. 7). To examine whether PlexinB1 activates the GTPase activity of Ran directly, purified cytoplasmic PlexinB1 protein of WT sequence (cytoPlexinB1-WT) or a mutant form of PlexinB1 with a non-functional GAP domain [23] (cytoPlexinB1-RA) were used in direct GTPase activating proteins (GAP) activity assays according to the method of Webb et al. [40] which measures the release of inorganic phosphate from the small GTPase over time (Supplementary Fig. 8A, B). CytoPlexinB1-WT protein had a similar level of GAP activity for RanGTP as RanGAP1 (Fig. 5A). PlexinB1 protein with mutations (R1677A, R1678A, R1984A) in the GAP domain, which displays no or very weak GAP activity [23] (cytoPlexinB1(RA)), lacked intrinsic GAP activity for RanGTP, as expected (Fig. 5A). The GAP activity of cytoPlexinB1-WT was dependent on the concentration of the RanGTP substrate protein (Fig. 5B) and on the concentration of cytoPlexinB1-WT (Fig. 5C). The initial rate of reaction for 68 μm PlexinB1 and 14.6 μm RanGTP was 0.189 μM[Pi]/s. Purified cytoplasmic PlexinB2 protein also demonstrated RanGAP activity at a slightly lower rate than PlexinB1 (0.12 μM[Pi]/s) for equivalent protein concentrations, while PlexinB3 had negligible GAP activity towards Ran (Fig. 5D). The GAP activity of cytoPlexinB2-WT was dependent on the concentration of the RanGTP substrate (Supplementary Fig. 8C). These results demonstrate that PlexinB1 and PlexinB2 act as GTPase activating proteins for Ran.

### RanGTP interacts with PlexinB1 and PlexinB2

**Fig. 6:**
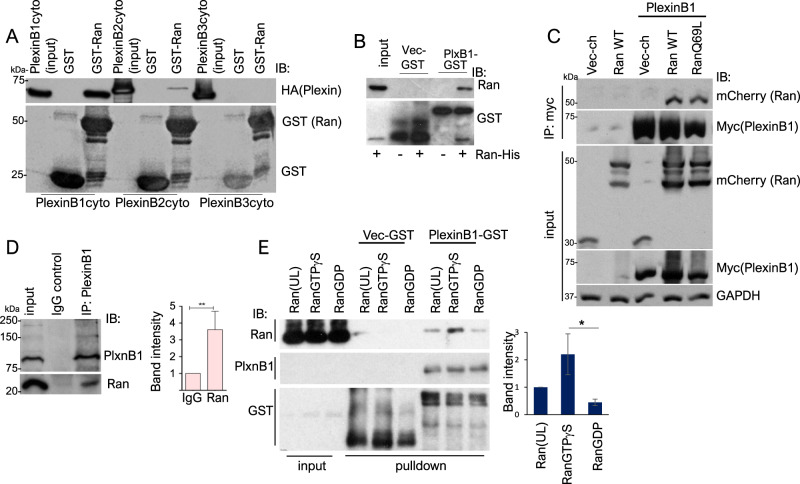
RanGTP interacts with PlexinB1 and PlexinB2. **A** PlexinB1 and PlexinB2 interact with Ran-GST. HEK293 cells were transiently transfected with HA-tagged cytoPlexinB1, cytoPlexinB2 or cytoPlexinB3. After 24 h, cells were lysed and incubated with GST or Ran-GST immobilised to glutathione-sepharose beads, and bound proteins were detected by immunoblotting with the anti-HA antibody. **B** Ran-His protein interacts with PlexinB1 directly. Purified His-tagged Ran(WT) protein was incubated with GST-cytoPlexinB1 or GST-empty vector. Bound proteins were analysed by immunoblotting with anti-Ran antibody. **C** Ran co-immunoprecipitates with full-length PlexinB1. COS7 were co-transfected with plasmids encoding mCherry-tagged Ran(WT), Ran(Q69L) or vector control and myc-tagged PlexinB1 (full length) or vector control. After 20 h, cell lysates were incubated with anti-myc-antibody bound to agarose for 2 h. Immunoprecipitated proteins were analysed by immunoblotting with indicated antibodies. **D** Interaction between endogenous Ran and endogenous PlexinB1. Precleared LNCaP cell lysates were incubated with either 1–3 µg of anti-mouse Ran antibody or IgG mouse (control) for 3 h, then with protein G agarose for a further 1 h. Co-immunoprecipitated and total endogenous proteins were detected using anti-PlexinB1 or anti-Ran antibody. Graph shows densitometry analysis of endogenous co-immunoprecipitation (*n* = 3) **p < 0.001, Student’s *t*-test. **E** PlexinB1 interacts with GTP-bound Ran preferentially. Purified Ran-His protein was labelled with GTPγS or GDP or left unlabelled (UL), then incubated with GST-cytoPlexinB1 or GST-empty vector. Bound proteins were analysed by immunoblotting with anti-Ran antibody. Graph shows densitometry analysis of pulled-down proteins (*n* = 3) *p < 0.05, Student’s *t*-test.

Immunoprecipitation experiments were next performed to establish whether PlexinB1 and B2 regulate RanGTP levels by direct physical interaction with Ran. PlexinB1 and PlexinB2, but not PlexinB3, were found to interact with Ran in pull-down assays using GST-Ran (Fig. 6A, Supplementary Fig. 9A). The results showed a preferential interaction of GST-Ran for cytoPlexinB1(WT) compared to cytoPlexinB2. Furthermore, GST-cytoPlexinB1(WT) pulled down purified Ran-His protein, demonstrating a direct interaction between the two proteins (Fig. 6B). Consistent with these results, Ran was found to co-immunoprecipitate with PlexinB1 in lysates of Cos7 cells co-expressing either full-length PlexinB1(WT) (Fig. 6C) or cytoPlexinB1(WT) (Supplementary Fig. 9) and Ran(WT) or RanQ69L. This association was further confirmed by the co-immunoprecipitation of endogenous Ran with endogenous PlexinB1 in HeLa cells (Fig. 6D).

Ran-GST pull-down assays using various PlexinB1 deletion constructs demonstrated that the Rho binding domain (RBD) of PlexinB1 is required for the interaction of Ran with PlexinB1 (Supplementary Fig. 9), while mutation of a highly conserved Proline residue (Pro1851) to Glycine in the RBD of PlexinB1 reduced Ran binding (Supplementary Fig. 9). These results suggest that Ran binds to the RBD region of cytoplasmic PlexinB1.

To establish if the binding of Ran to PlexinB1 is dependent on whether Ran is bound to GTP or GDP, pull-down experiments were performed using purified Ran-His protein labelled with GTPγS (a non-hydrolysable form of GTP) or GDP. PlexinB1 interacted with RanGTP preferentially (Fig. 6E). Together, these results show that PlexinB1 and B2 interact with Ran directly in a GTP-dependent manner.

### Subcellular localisation of PlexinB1, Sema4D and Ran

**Fig. 7:**
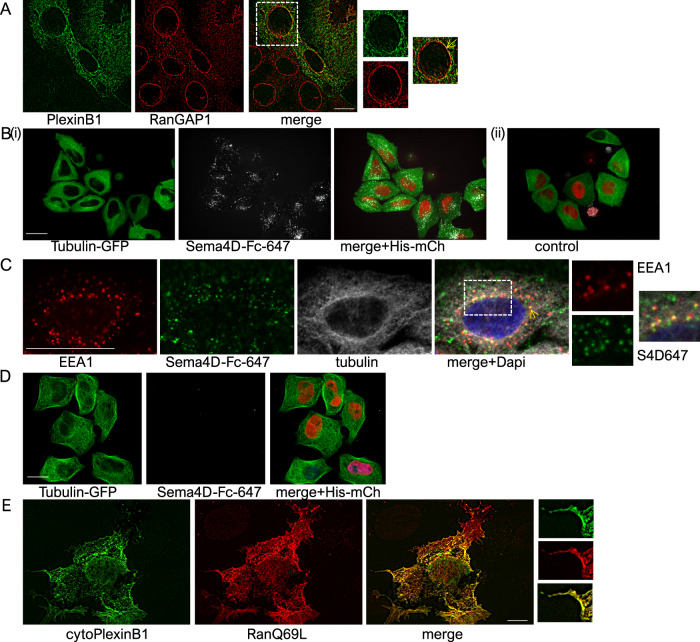
Subcellular localisation of PlexinB1, Sema4D and Ran. **A** High resolution (N-SIM-S Super Resolution Microscope) image of COS7 cells expressing PlexinB1 (full-length)-myc and stained for RanGAP1 (red) and myc (green). Arrow shows perinuclear RanGAP1 and PlexinB1 (magnification ×100; scale bar = 10 μm). **B** Uptake of exogenous Sema4D-Fc labelled with Alexa647 into the cell. Representative image of HeLa cells expressing GFP-tubulin and mCherry-Histone treated with Sema4D-Fc-647 (i) or vehicle control (ii) for 2 h. Single slice of a z-stack through the centre of the cell, showing uptake of Sema4D into the cell (×60 magnification scale bar = 25 μm). **C** HeLa cells expressing GFP-tubulin, treated with Sema4D-Fc-647 for 2 h, fixed and stained for EEA1 and Dapi. Arrow shows the colocalization of EEA1 and Sema4D-Fc-647 in the cell (×60 magnification scale bar = 25 μm). **D** Inhibition of Sema4D-Fc-647 uptake with Dynasore treatment. Representative image of HeLa cells expressing GFP-tubulin and mCherry-Histone treated with Sema4D-Fc-647 for 2 h and with Dynasore for 30 min. Single slice of a z-stack through the centre of the cell (×60 magnification scale bar = 20 μm). **E** High resolution (N-SIM-S) image of COS7 cells expressing cytoPlexinB1-myc (green) and RanQ69-cherry (red) (Pearson’s coefficient 0.835); scale bar = 10 μm.

PlexinB1 activation influences the nuclear/cytoplasmic transport of nuclear receptors [33, 34], which is regulated by the RanGTP/GDP cycle [5, 6]. These findings led us to speculate that PlexinB1 may have a role within the cell in addition to its function on the plasma membrane. We found that PlexinB1 is expressed in the cytoplasm as well as on the cell membrane and around the nuclear envelope, partially co-localising with RanGAP1 (Fig. 7A, Supplementary Fig. 10A, B) in interphase cells. Furthermore, Sema4D-Fc, the ligand for PlexinB1, is imported into the cell following treatment with the fluorescently tagged protein (Fig. 7B, Supplementary Fig. 10C, D, Supplementary Video 1) and colocalises with the endosome marker EEA1 (Fig. 7C). Treatment of cells with an inhibitor of dynamin-dependent endocytosis (Dynasore) attenuates internalisation of Sema4D-Fc-647 (Fig. 7D), suggesting that Sema4D enters the cell by clathrin-mediated endocytosis. Furthermore, Sema4D-Fc colocalises with full-length PlexinB1 (Supplementary Fig. 10E). These results show that Sema4D is taken up by the cell into the cytosol and supports a role for Sema4D/ PlexinB1 within the cell.

RanQ69L, a mutant form of Ran which lacks GTPase activity, is found predominantly in the cytoplasm of interphase cells—Ran employs NTF2 as a carrier to facilitate re-entry into the nucleus, and NTF2 is specific for the GDP-bound form of Ran [16]. RanGTP and PlexinB1 partially colocalise in the cytoplasm and plasma membrane of Cos7 cells co-transfected with RanQ69L-cherry and cytoPlexinB1-myc (Fig. 7F, Pearson’s coefficient 0.835; Supplementary Fig. 10E, Pearson’s coefficient 0.846) or full-length PlexinB1-myc (Supplementary Fig. 10G, Pearson’s Coefficient 0.879).

## Discussion

The findings of this study provide evidence for a novel function for PlexinB1 and PlexinB2 as RanGAPs. Consistent with a role for plexins in the regulation of Ran, both proteins have been shown to share a role in the regulation of nucleocytoplasmic trafficking, mitotic spindle orientation [41, 42] and retrograde axonal endosomal transport [43, 44]. Sema4D/3C -induced translocation of GR to the nucleus is dependent on an intact NLS [34], pointing to the involvement of Ran and importins in this process. The Ran binding protein, RanBP9, interacts with PlexinA1 and depletion of RanBP9 leads to reduced Sema3A-induced axonal outgrowth [45]. The activity of B-type Plexins as RanGAPs may partially explain the increase in nuclear trafficking of AR and GR [33, 34] and the increase in CRPC growth with Sema3C expression [32].

PlexinB1 is a GAP for Rap [29] as well as Ran. The process of GTP hydrolysis by small GTPases involves the nucleophilic attack by a water molecule on the terminal γ phosphate of the GTP substrate and cleavage of the phospho-monoester bond to produce GDP and inorganic phosphate. For most small GTPases, including Ran, the water molecule is positioned by interaction with a conserved glutamine residue (Gln61 in Ras, Gln69 in Ran), immediately following the DxxG motif in the switch II region of the small GTPase. Rap, in contrast, utilises a Gln residue three residues after the DxxG motif. Plexins and many other GAPs possess a crucial arginine residue known as an ‘arginine finger’, which interacts with and stabilises the leaving γ phosphate group in GTP. In contrast, the canonical GAPs for both substrates utilise different catalytic mechanisms. RapGAP [46] utilises an asparagine residue in place of the Arg finger (known as an ‘Asn thumb’), while RanGAP stabilises the catalytic position of Gln69 in Ran in the absence of either the Arg finger or Asn thumb. Hence, plexins act by a different mechanism from either RapGAP or RanGAP on their respective substrates.

RanGAP1, the only GAP for Ran demonstrated up till now, is located around the nuclear envelope by association with RanBP2 in interphase cells [47], while PlexinB1, a transmembrane receptor, localises predominantly to the plasma membrane. In their function as RanGAPs, plexins may facilitate the direct regulation of the Ran system by extracellular cues at the plasma membrane, thereby serving a function distinct from that of RanGAP1.

Plexins may have a role in the release and consequent activation of cargo proteins bound to exportins at the cell membrane. RanGTP promotes the binding of NES-containing cargo proteins to exportin in the nucleus; disassembly of this cargo-exportin-RanGTP complex occurs upon GTP hydrolysis catalysed by a RanGAP. Plexins, acting as RanGAPs at the plasma membrane, may stimulate the localised release and consequent activation of cargo from this complex in response to semaphorin stimulation. RanGTP associates with RhoA and targets RhoA in the plasma membrane, where it is released upon RanGTP hydrolysis and the interaction of RhoA with RanGTP is disrupted by RanBP1 overexpression [48]. PlexinB1 may act to release RhoA from this RanGTP-RhoA complex at the plasma membrane by catalysing the conversion of RanGTP to RanGDP, freeing RhoA to perform its function.

Plexins may also function as RanGAPs within the cytoplasm, liberating importins from RanGTP-importin complexes to allow their binding to more cargo. Our results have demonstrated that PlexinB1 is expressed in the cytoplasm and around the nuclear envelope and that Sema4D is internalised by endocytosis, revealing a capacity to signal from endosomal receptors in the cell in a similar way to many other transmembrane receptor proteins such as Met [49].

These findings are clinically significant as ligand-independent nuclear translocation of AR and GR promotes resistance to ADT in the treatment of prostate cancer, currently a major clinical problem. PlexinB1 activation promotes nuclear trafficking of both receptors and increases CRPC growth in mouse models. We have found that Sema4D-induced nuclear translocation of AR is dependent on Ran signalling and the GAP domain of PlexinB1, suggesting that PlexinB1/B2 contribute to ADT resistance through their function as RanGAPs. AR is imported into the nucleus in the absence as well as the presence of androgens, where it is polyubiquitinated and degraded [50]. In CRPC, AR degradation in the nucleus is reduced, allowing the transcription of AR-responsive genes [50]. Androgen-independent and androgen-dependent import of AR relies on the RanGTP/GDP system [5]. Blocking Sema4D/4C-PlexinB1/B2 signalling may therefore be of benefit to the treatment of ADT resistance in late-stage prostate cancer.

An in-balance in the plexin-Ran signalling pathway may also, in part, account for the well-documented role of plexins in many cancers.

## Methods

### Cell culture and transfection

All cell lines were from ATCC (LGC standards, Middlesex, UK) and were STR typed to confirm their identity. Cos7, MCF7 and HEK293 cells were grown in Dulbecco’s modified Eagle’s medium (DMEM) containing 10% foetal calf serum (FCS) and penicillin-streptomycin (Invitrogen). LNCaP cells were grown in RPMI medium with 10% FCS (Invitrogen). Cos7, or HEK293 cells were transiently transfected with the indicated plasmids using Lipofectamine 2000 or 3000 (Invitrogen) as per manufacturer’s instruction.

### SiRNA

Short interfering RNA (siRNA) sequences targeting PlexinB1, PlexinB2 and PlexinB3 were obtained from Dharmacon. Plexin expression was knocked down using two different siRNA oligos against PlexinB1: siPlexin B1-1 (*GAGAGGAGCCGACUACGUA*), siPlexin B1-2 (*GCAGAGACCUCACCUUUGA*), against PlexinB2: siPlexin B2-1 (*GCAACAAGCUGCUGUACGC*) siPlexin B2-2 (*UGAACACCCUCGUGGCACU*), and against PlexinB3: Plexin B3-1 (*CAAAGUGACUGACCUGUGA*), Plexin B3-2 (*AAACGGACCCUGAAUGAUA*) (siGenome Dharmacon, Lafayette, CO, USA), together with siGENOME non-targeting siRNA pool (Dharmacon) as control. 25 Nm of siRNA was transfected using Dharmafect (Dharmacon) or Oligofectamine (Thermo Fisher) according to manufacturers’ instructions. Post 48 h, cells were fixed for immunofluorescence or after 72 h for time-lapse video microscopy or lysed for biochemical analysis.

### Expression vectors and site-directed mutagenesis

pcDNA3-PlexinB1 full-length-myc (PlexinB1-myc), pcDNA3-Plexin B1 cytoplasmic domain-Myc (cytoPlexinB1-myc), PlexinB1(RA)-myc (R1677A, R1678A, R1984A) and cytoPlexinB1(RA)-myc were kind gifts of Dr I Oinuma (Kyoto, Japan). pmCherry-C1-RanQ69L was a gift from Jay Brenman (Addgene plasmid # 30309; http://n2t.net/addgene:30309; RRID:Addgene_30309), pcDNA-Ran-T24N-Mrfp1-polyA from Yi Zhang (Addgene plasmid # 104561; http://n2t.net/addgene:104561; RRID:Addgene_104561), pcDNA-RanWT-Mrfp1-polyA from Yi Zhang (Addgene plasmid # 59750; http://n2t.net/addgene:59750; RRID:Addgene_59750), GFP-RelA from Warner Greene (Addgene plasmid # 23255; http://n2t.net/addgene:23255; RRID:Addgene_23255) [51–53], Px-RCC1-HA from Patrizia Lavia (Addgene plasmid # 106938; http://n2t.net/addgene:106938; RRID:Addgene_106938) [54].

pGex-GST-cytoPlexinB1, pGex-GST-cytoPlexinB2 and pGex-GST-cytoPlexinB3 were generated by subcloning from the corresponding full-length Plexin pcDNA3 plasmids using NEBuilder® HiFi DNA Assembly Master Mix (E2621S NEB), using the primers: pGex-GST-cytoPlexinB1: *ATCGGATCTGGTTCCGCGTGGCAGGAGGAAGAGCAAGCAG* and *GTCAGTCACGA TGCGGCCGCTCCTATAGATCTGTGACCTTGTTTTC;* pGex-GST-cytoPlexinB2: *ATCGGATCTGG TTCCGCGTGGGTGCTACTGGAGGAAGAGC* and *GTCA GTCACGATGCGGCCGCTCAGTGAC CTTGTTCTCCAG;* pGex-GST-cytoPlexinB3*: ATCGGATCTGGTTCCGCGTGGGAGGCACAAGA GCAAGCAG* and *GTCA GTCACGATGCGGCCGCTCTCACAGGTCAGTCACTTTG*. Pet28-cytoPlexinB1(WT) and Pet28-cytoPlexinB1(RA) were generated by subcloning from PlexinB1(WT)-myc and PlexinB1(RA)-myc into Pet28a using the primers: *ATGGTCCGCGGAT CCGAATTCAGGAGGAAGAGCAAGCAG* and *TCGAGTGCGGCCGCAAGCTTGCTATAGATCTGTGAC CTTGTTTTC*.

PlexinB1 deletion constructs: cytoPlexinB1 ∆RBD; cytoPlexinB1∆5’; cytoPlexinB1∆3’; and cytoPlexinB1(P1851G) were generated from cytoPlexinB1-HA using Q5^®^ Site-Directed Mutagenesis Kit (E05545, NEB) and the following primers: cytoPlexinB1∆RBD: CTCACCAAGCATGTGCTC and GTA CTCCACATCCTCTCTG, cytoPlexinB1∆3’: GTGGAAAACAAGGTCACAGATCTATAGAGG AG and GCA GGG GAC GAG GGC CAC, cytoPlexinB1∆5’: CGTCCCTGACCTTGAATG and GTACTCCAC ATCCTCTCTG, cytoPlexinB1(P1851G): GGCCCTCGTTCGGATGCCTCACCA and ACAGTTGCTCCATCTGGG.

pGex-GST-Ran(WT), pGex-GST-Ran(Q69L) and pGex-GST-Ran(T24N) were generated by subcloning from pcDNA-RanWT-Mrfp1-polyA, pmCherry-C1-RanQ69L and pcDNA-Ran-T24N-Mrfp1-polyA respectively, using the primers: *ATCGGATCTGGTTCCGCGTGGGATGGCTGCGCAGGGAGAG* and *GTCAGTCACGAT GCGGCCGCTCCAGGTCATCATCCTCATCCGG*. Pet28-Ran(WT) was generated by subcloning from pcDNA-RanWT-Mrfp1-polyA into Pet28a using the primers: *ATGGTCCGCG GATCCGAATTCATGGCTGCGCAGGGAGAG* and *TCGAGTGCGGCCGCAAGCTTGCAGGTC ATCATCCTCATCCG*. pEGF-GFP-Rnd2 and pCMV-FLAG-Rnd2 were made as described previously (Azzarelli et al.) [55]. All nucleotide changes were verified by DNA sequencing (Eurofins-MWG, UK).

The Ran binding domain of RanBP1 (amino acids 103–241) was cloned into pGex using the primers: ATCGGATCTGGTTCCGCGTGCAGTTTGAGCCAATAGTTTC and TCGTCAGTCAGTCACGATGCCTCTTCGA TCTCTTTCCTG to generate the plasmid RanBP1(RBD)-GST.

### Immunocytochemistry

Cells grown on coverslips were fixed (4% paraformaldehyde), permeabilized (0.2% triton), and stained by immunofluorescence. The following primary antibodies were used: mouse anti-Myc antibody clone 9E10 (13-2500 Sigma-Aldrich (1:1000)), mouse anti-Flag (F1804, Sigma (1:1000)), rabbit polyclonal anti-Myc (C3956, Sigma-Aldrich (1:1000)), goat anti-PlexinB1 (AF349, R&D Systems (1:500)), mouse anti-RanGAP1 (NBP2-0262, Novus Biologicals (1:500)), rabbit anti-Ran (4462, CST (1:800)), mouse anti-Ran (240902, Cell-Biolabs (1:800)), Mouse anti-β-tubulin (clone DMIA, MABT205, Sigma-Aldrich (1:1000)), rabbit anti-pericentrin (ab22084, Abcam (1:800)), rabbit anti-EEAI (2411, CST (1:500)), rat anti-RelA (MAB50781, R&D (1:500)). Alexa-Fluor488, Alexa-Fluor555, Alexa-Fluor594, or Alexa-Fluor647- conjugated secondary antibodies and Alexa-Fluor 633 Phalloidin were from Life Technologies (A21235, A21082, A21448, A21246, A266, A11034, A32814). Coverslips were mounted with Prolong Gold Antifade mountant (8961S Life Technologies). Images were taken at ×63 magnification using a Zeiss LSM510 confocal microscope or at 40× or 60× magnification using a Nikon A1 inverted confocal with a spectral detector or a Nikon Inverted spinning disk confocal microscope and at 100× with a Nikon N-SIM-S Super Resolution Microscope.

### Sema4D treatment for immunocytochemistry studies

In experiments to assess the internalisation of Sema4D, Sema4D-Fc (5235-S4B, Bio-Techne) was labelled with Alexa647 (Alexafluor microscale labelling kit, A30009, Thermo Fisher), following manufacturer’s instructions. HeLa cells expressing GFP-tubulin and mCherry-Histone plated in chamber slides were incubated with Sema4D-Fc-Alexa647, fixed, stained for EEA1 (2411, CST) and imaged using a Nikon Inverted spinning disk confocal microscope at 60× magnification and z stacks made.

Cells were also treated with 80 mM Dynasore (120287002, Sigma) or vehicle control for 30 min with or without Sema4D-Fc-647 (2 h, 2 μg/ml), then fixed and stained with DAPI.

### Nuclear translocation of androgen receptor

PC3 cells were transfected with AR-Flag variant 2 and empty vector (pmCherry), pmCherry-RanWT pmCherry-RanQ69L or pmCherry-RanT24N, together with Fc empty vector or Sema4D-Fc. Transfected cells were serum starved overnight, fixed, and stained for AR with anti-Flag-tag antibody, with anti-Fc antibody and with DAPI.

In separate assays, serum-starved PC3 cells were transfected with AR-Flag variant 2 and pmCherry or pmCherry-RanQ69L and treated with Sema4D-Fc (2 μg/ml, Bio-techne), DHT (Sigma) or vehicle control for 1.5 h or 1 h respectively.

Cells were imaged at x60 using a Nikon Inverted spinning disk confocal microscope, and cells transfected with all constructs were scored blind for (1) Nuclear staining of AR exceeded cytoplasmic AR (N > C), (2) Nuclear staining of AR was equal to cytoplasmic AR (C = N), (3) Nuclear staining of AR was less than cytoplasmic AR (C > N). More than 50 cells were scored per condition per experiment (*n* = 3).

In separate assays, PC3 cells were transfected with AR-flag and Vector-myc or full-length PlexinB1(WT)-myc or PlexinB1(RA)-myc. Twenty-four hours after transfection, serum-starved cells were treated with PBS or Sema4D-Fc (2 μg/ml, Bio-techne) for 1.5 h before fixing and immunocytochemistry.

### Collapse assay

Cos7 cells were plated (1 × 10^5^ cells/ml) onto glass coverslips, and 24 h later, cells were transiently transfected with plasmids encoding for empty vector (pmCherry) or co-transfected with myc-PlexinB1 together with pmCherry-RanWT, pmCherry-RanQ69L, pmCherry-RanT24N or pmCherry using Lipofectamine 2000 as per manufacturer’s instruction. After 18 h, cells were fixed for 15 min with 3.7% paraformaldehyde and permeabilised for 5 min with 0.2% Triton X-100. Coverslips were incubated for 1 h with rabbit anti-myc antibody to detect myc-tagged Plexin B1. Mouse anti-β-tubulin (clone DMIA) was used to detect microtubules. AlexFluor 488 antibody conjugated donkey anti-rabbit antibody identified PlexinB1 expressing cells, and AlexFluor 647 antibody conjugated donkey anti-mouse antibody revealed microtubular organisation. Ran-expressing cells were visualised using mCherry. After extensive washing, coverslips were mounted on glass microscope slides, and images were generated using Eclipse Ti-E inverted confocal microscope (Nikon) DU-897X-3430 camera (Andor) with PlanFluor x40/1.3 NA oil objective. ‘Collapsed’ cells were defined as cells with a contracted cell body and with one or more extensions of greater length than the cell body.

### Ran nuclear/cytoplasmic ratio

The intensity of staining for Ran in the cytoplasm and nucleus following transfection of HeLa cells expressing GFP-tubulin or MCF or PC3 cells with non-silencing siRNA or siRNA to PlexinB1 was measured using ImageJ (NIH) using DAPI staining to outline the nucleus and tubulin or Plexin staining to identify the cytoplasm. The ratio of nuclear to cytoplasmic staining for each cell was calculated (*n* = 4, A minimum of 284 cells were scored per treatment).

### Time-lapse microscopy

For videos of Sema4D-Fc-647 endocytosis, HeLa cells expressing GFP-tubulin and mCherry-Histone plated in chamber slides were incubated with Sema4D-Fc-Alexa647 in FluoroBrite DMEM Media (Thermo Fisher Scientific) with 10% serum, 1 mM sodium pyruvate and 2 mM glutamine and imaged using a Nikon Inverted spinning disk confocal microscope at ×40.

### GST pull-downs

*Rosetta*™ Competent Cells (Novagen) transformed with Pgex-GST-Ran(WT), Pgex-GST-Ran(Q69L) and Pgex-GST-Ran(T24N), Pgex-GST-PlexinB1(cyto), Pgex-GST-PlexinB2(cyto), Pgex-GST-PlexinB3(cyto) or RanBP1(RBD)-GST were grown overnight in Luria Broth and glutathione-S-transferase (GST) fusion protein expression induced with IPTG 1 mM for 3 h. Pelleted cells were lysed with lysis buffer (B-PER Bacterial Protein Extraction Reagent, ThermoFisher), lysozyme (100 μg/ml), PMSF (1 mM, Sigma), protein inhibitor cocktail (ThermoFisher), DNAse (5U/ml), at room temperature for 15 min, then on ice for 15 min, then spun for 30 min. The cleared lysate was incubated with Glutathione Sepharose 4B (17075601, GE Healthcare) for 2 h at 4 °C then washed. Recombinant GST was used as a control.

Transfected COS7 cells were washed and lysed in lysis buffer (1% Triton X-100, 20 mM Tris-HCl [Ph 8], 130 mM NaCl, 10 mM NaF, 1% aprotonin, 10 μg/ml leupeptin, 1 mM dithiothreitol (43816, DTT), 0.1 mM Na_3_VO_4_ and 1 mM phenylmethylsulfonyl fluoride (PMSF)). Insoluble material was removed by centrifugation, and the cell lysates were combined for 2 h at 4 °C with the recombinant GST-fusion proteins bound to glutathione-sepharose beads. Beads were washed extensively with lysis buffer before the proteins were eluted in the Laemmli sample buffer. Bound proteins were analysed by immunoblotting. GFP-Trap agarose beads (gta-20) were supplied by ChromoTek, mouse anti-FLAG agarose (A2220) from Sigma-Aldrich, mouse anti-HA antibody bound to agarose clone HA.7 (A2095) from Sigma-Aldrich.

### Immunoprecipitations

Cos7 cells co-transfected with indicated plasmids were lysed with ice-cold cell lysis buffer (20 mM Tris-HCl, Ph 8, 130 mM NaCl, 1% Triton X-100, 1 mM DTT, 10 mM NaF, 1 mM phenylmethylsulfonyl fluoride, 10 µg/ml aprotinin, 10 µg/ml leupeptin, 0.1 mM sodium orthovanadate). After centrifugation, the supernatants were incubated with 20 µl of anti-myc antibody bound to agarose or anti-HA antibody bound to agarose beads (Sigma) for 2 h at 4 °C with rotation. The beads were washed extensively with lysis buffer, and bound proteins were analysed by SDS-PAGE and immunoblotting.

### Endogenous co-immunoprecipitation

LnCaP cells were lysed and scraped in cold lysis buffer (20 mM Tris-HCl, Ph 8, 130 mM NaCl, 1% Triton X-100, 1 mM DTT, 10 mM NaF, 1 complete EDTA-free protease cocktail tablet (Roche), Phosphatase cocktail inhibitors II and IV (Sigma)). Clarified supernatant was kept after spinning at 13.2 rpm for 15 min, and a total of 40 µl was taken to determine the expression of endogenous protein. The clarified supernatant was precleared by incubating with 30 µl of protein G agarose fastflow (Sigma) for 45 min with rotation. The supernatant was spun for approximately 1 min at 13.2 rpm and removed to a cold Eppendorf tube. Anti-mouse Ran antibody was added to a concentration of 1–3 µg and rotated for approximately 3 h at 4 °C. After 3 h, the supernatant was incubated with 30 µl of protein G agarose Fastflow for an extra 1 h, washed extensively with lysis buffer containing 250 mM NaCl (spun at 3000 rpm for 1 min). Laemmli sample buffer containing DTT was added to the bound protein and boiled before being subjected to SDS-PAGE analysis.

### Immunoblotting

For immunoblotting, proteins were resolved by SDS-PAGE and transferred to a nitrocellulose membrane (Schleicher and Schuell). Membranes were blocked with TBS (20 mM Tris-HCl [Ph 7.6], 137 mM NaCl) containing 5% non-fat dried milk and 0.05% Tween 20. Bound antibodies were visualised with horseradish peroxidase-conjugated goat anti-immunoglobulin G antibodies and enhanced chemiluminescence (ECL; Amersham Pharmacia Biotech). The following primary antibodies were used: Rabbit anti-mCherry (Abcam, ab 183628), Mouse anti-GAPDH (MAB374, Millipore, now Merck), rabbit polyclonal anti-FLAG (M2, F-7425 Sigma-Aldrich), mouse anti-Myc antibody clone 9E10 (13-2500 Sigma-Aldrich), rabbit polyclonal anti-Myc antibody (Sigma-Aldrich), rabbit anti-GFP antibody (A-11122 Sigma-Aldrich), Goat polyclonal anti-GST (GE27-4577, Merck), rat monoclonal anti-HA antibody clone 3F10 (11867 423001, Roche Applied Science), anti-PlexinB1 (PP1841, ECM Biosciences), anti-PlexinB2 (AF5329, R&D Systems), anti-PlexinB3 (AF4958, R&D Systems). Secondary antibodies used for Western blotting: horseradish peroxidase linked (HRP) polyclonal goat anti-mouse IgG (P0447), goat anti-rabbit IgG (P0448), rabbit anti-goat IgG (P0449) (Agilent) or goat anti-rat IgG (sc-2006) (Santa Cruz Biotechnology).

### Loading of Ran-His with GTPγS or GDP

Purified Ran-His protein was loaded with GTPγS or GDP in the following reaction: 3.0 μM Ran-His was incubated with 0.5 mM GTPγS or GDP at 30 °C for 10 min in 20 mM Tris-HCl Ph 7.5, 100 mM NaCl, 5 mM EDTA, 2 mM DTT. Then, 50 mM MgCl_2_ was added to stop the reaction on ice. The labelled protein was used in pull-down assays with Vec-GST or PlexinB1-GST glutathione Sepharose beads.

### Ran activation assay

Levels of RanGTP were assessed by immunoprecipitation using RanBP1(RBD)-GST fusion proteins bound to glutathione-sepharose beads, which selectively isolate the active form of Ran (RanGTP) in the lysis Buffer: 25 mM HEPES, Ph 7.5, 150 mM NaCl, 1% NP-40, 10 mM MgCl2, 1 mM EDTA, 2% Glycerol, 10 mM NaF, protease inhibitor tablet (Roche), followed by western blot analysis with anti-Ran antibody. Cells were treated with siRNA for 72 h or Sema4D-Fc or control conditioned medium for 15 min prior to pull down. For rescue experiments, cells were transfected with RCC1-HA, or vector-HA, then treated with Sema4D or control conditioned medium the following day.

### GAP activity assay

Protein for the assay was extracted from *Rosetta*™ Competent Cells (70954, Novagen) expressing Pet28-Ran(WT), Pet28-cytoPlexinB1(WT) or Pet28-cytoPlexinB1(RA) Pet28-cytoPlexinB2, Pet28-cytoPlexinB3 or Pet28-RanGAP1 using a Ni-NTA Fast Start Kit (30600, Qiagen) according to manufacturers’ instructions. The proteins were purified on a Sephadex G-25 PD10 column (17085101, GE Healthcare). Aliquots were snap-frozen and stored at −70 °C.

The GAP activity was measured using the photometric assay according to the method of Webb et al. [40], in which the inorganic phosphate released upon hydrolysis of GTP is coupled to a reaction catalysed by purine nucleoside phosphorylase (PNP) using methylthioguanosine (MESG) as substrate. The enzymatic conversion of MESG to its guanine base form and ribose–phosphate was monitored spectrophotometrically by an increase in absorbance at 360 nm.

The absorbance at 360 nm of a reaction mixture containing 20 mM Tris-HCL Ph 7.6, 1 mM EDTA, 10 mM ammonium sulphate, 0.2 mM dithiothreitol (Sigma), 0.2 mM MESG (Berry & associates), 1.25U PNP (Sigma), 4 μM small GTPase and 0.5 mM GTP (Cytoskeleton Inc.). The reaction was initiated by the addition of the purified cytoPlexinB1(WT), cytoPlexinB1(RA), cytoPlexinB2, cytoPlexinB3 or RanGAP1 and the GAP-catalysed reaction was monitored continuously by the change in absorbance at 360 nm for 5 min at 10-s intervals. The linearity of response of absorbance to Pi concentration and the amount of Pi released during the reaction were calculated by use of a standard curve of absorbance at 360 nm vs a range of concentrations of KH_2_PO_4_, measured in the same assay buffer (Supplementary Fig. 8).

Values in the absence of Ran (control) were subtracted for each time point to control for spontaneous MESG decay and endogenous RanGTPase activity. Rap1B-His was obtained from Cytoskeleton Inc. Similar results were found for three different preparations of PlexinB1(WT) protein and three different preparations of PlexinB1(RA) protein made in parallel.

### Statistics

Statistical analysis was performed using Microsoft Excel or GraphPad. Error bars on graphs denote the average values ± standard error of the mean (SEM); statistical significance was determined by two-tailed paired Student’s *t*-test unless otherwise stated. All blots are representative of three or more independent experiments.

### Supplementary information

Supplementary video. Uptake of Sema4D into cell

Supplementary Figure 1

Supplementary Figure 2

Supplementary Figure 3

Supplementary Figure 4

Supplementary Figure 5

Supplementary Figure 6

Supplementary Figure 7

Supplementary Figure 8

Supplementary Figure 9

Supplementary Figure 10

Supplementary video

## Data Availability

All data generated or analysed during this study are included in this published article and its supplementary information files.
