# Supplementary material for: B-type Plexins promote the GTPase activity of Ran to affect androgen receptor nuclear translocation in prostate cancer
Source: Cancer Gene Ther. 2023 Aug 10;30(11):1513–23. doi: 10.1038/s41417-023-00655-6 (PMC10645588; doi:10.1038/s41417-023-00655-6)
Supplement: Supplementary file 2 — Supplementary Figure 1 [file 41417_2023_655_MOESM2_ESM.pptx]

## Slide 1
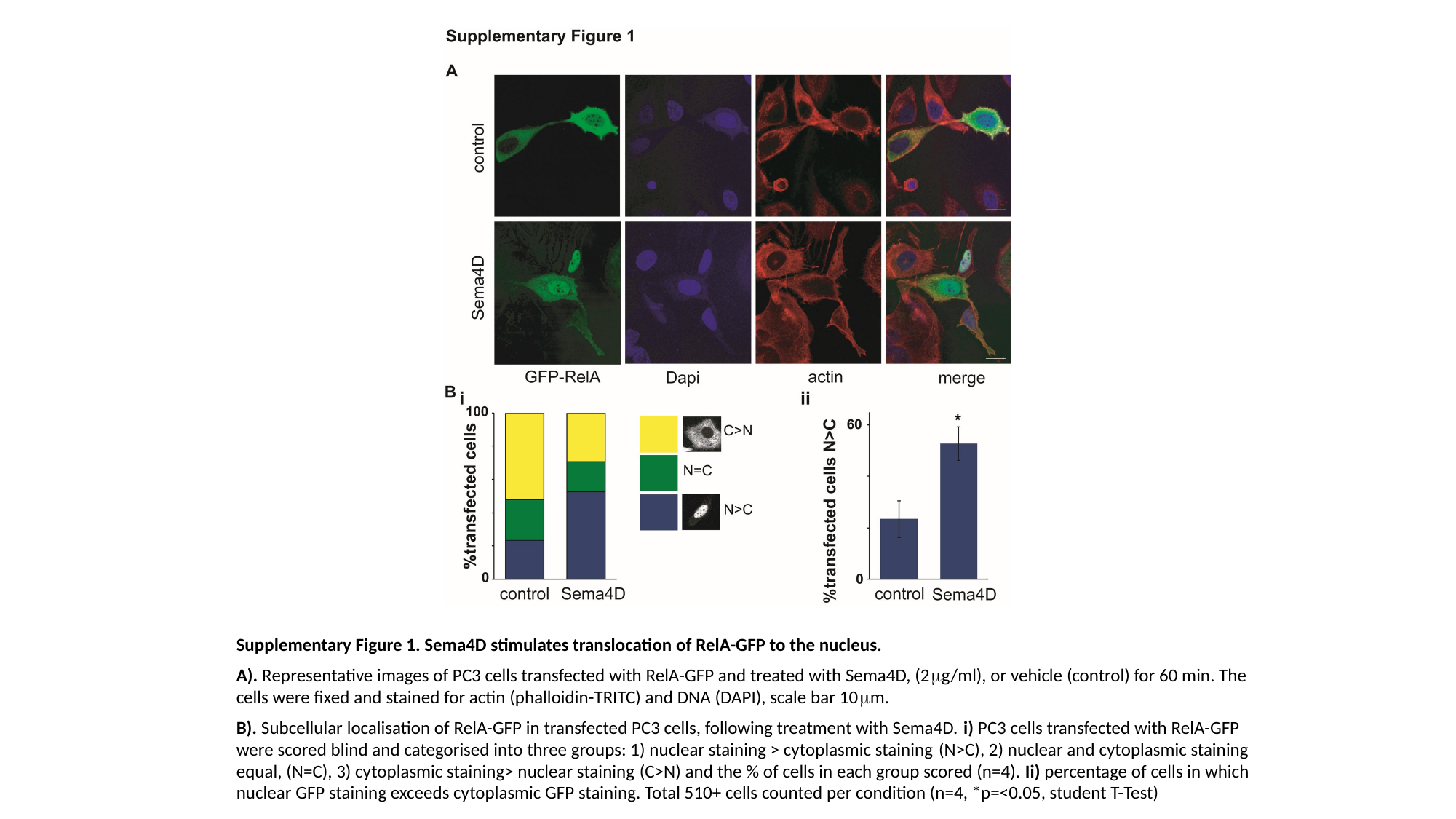

Supplementary Figure 1. Sema4D stimulates translocation of RelA-GFP to the nucleus.
A). Representative images of PC3 cells transfected with RelA-GFP and treated with Sema4D, (2mg/ml), or vehicle (control) for 60 min. The cells were fixed and stained for actin (phalloidin-TRITC) and DNA (DAPI), scale bar 10mm.
B). Subcellular localisation of RelA-GFP in transfected PC3 cells, following treatment with Sema4D. i) PC3 cells transfected with RelA-GFP were scored blind and categorised into three groups: 1) nuclear staining > cytoplasmic staining (N>C), 2) nuclear and cytoplasmic staining equal, (N=C), 3) cytoplasmic staining> nuclear staining (C>N) and the % of cells in each group scored (n=4). Ii) percentage of cells in which nuclear GFP staining exceeds cytoplasmic GFP staining. Total 510+ cells counted per condition (n=4, *p=<0.05, student T-Test)
