# Supplementary material for: B-type Plexins promote the GTPase activity of Ran to affect androgen receptor nuclear translocation in prostate cancer
Source: Cancer Gene Ther. 2023 Aug 10;30(11):1513–23. doi: 10.1038/s41417-023-00655-6 (PMC10645588; doi:10.1038/s41417-023-00655-6)
Supplement: Supplementary file 3 — Supplementary Figure 2 [file 41417_2023_655_MOESM3_ESM.pptx]

## Slide 1
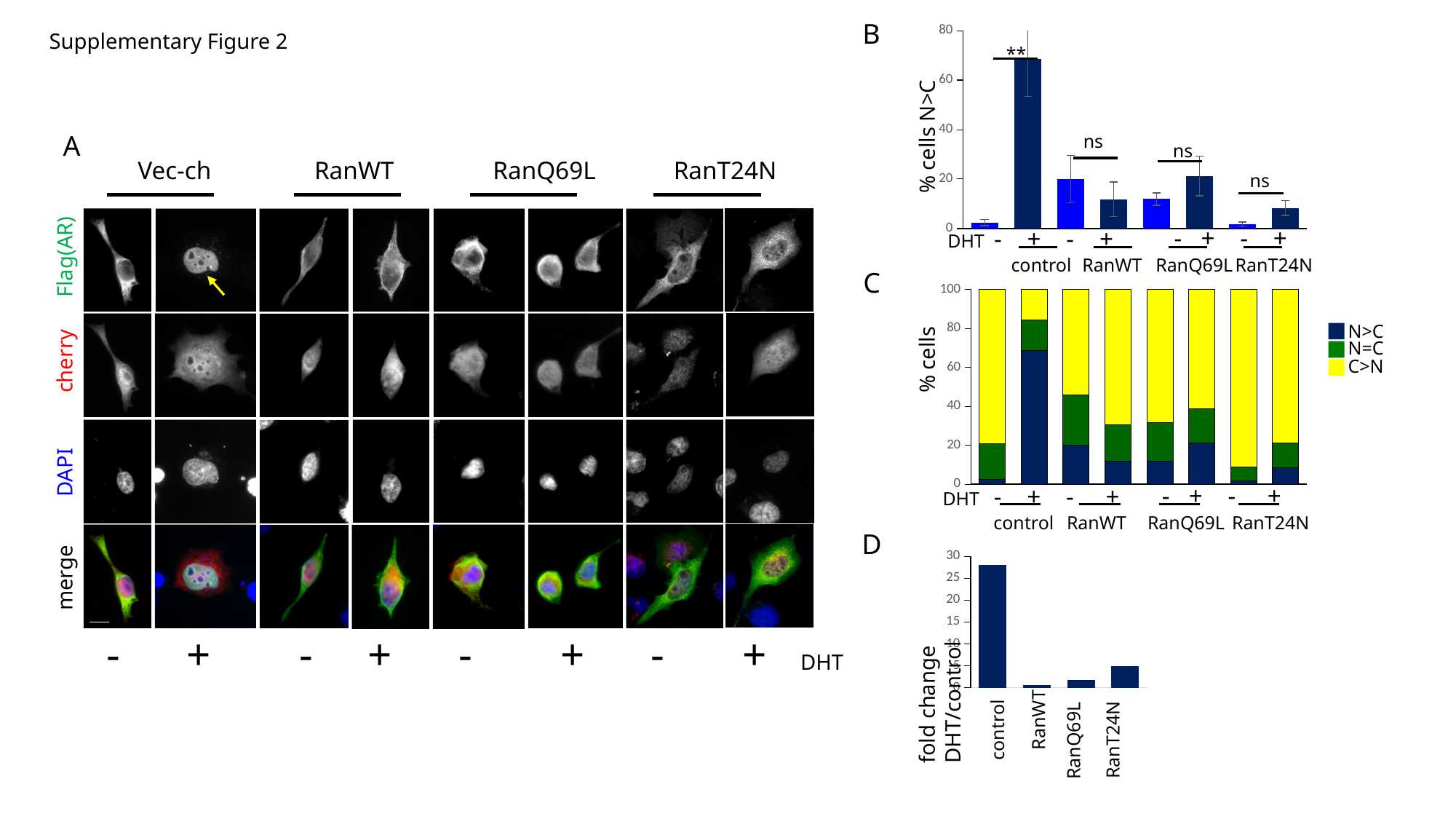

### Chart
| Category | |
|---|---|**
ns
ns
ns
- + - +
DHT - + - +
control
RanWT
RanQ69L
RanT24N
B
% cells N>C
C
### Chart
| Category | N>C | N=C | C>N |
|---|---|---|---|- + - +
 DHT - + - +
control
RanWT
RanQ69L
RanT24N
N>C
N=C
C>N
% cells
D
### Chart
| Category | |
|---|---|fold change DHT/control
RanWT
control
RanT24N
RanQ69L
Supplementary Figure 2
A
Vec-ch
RanWT
RanQ69L
RanT24N
Flag(AR)
cherry
DAPI
merge
- + - + DHT
- + - +

## Slide 2
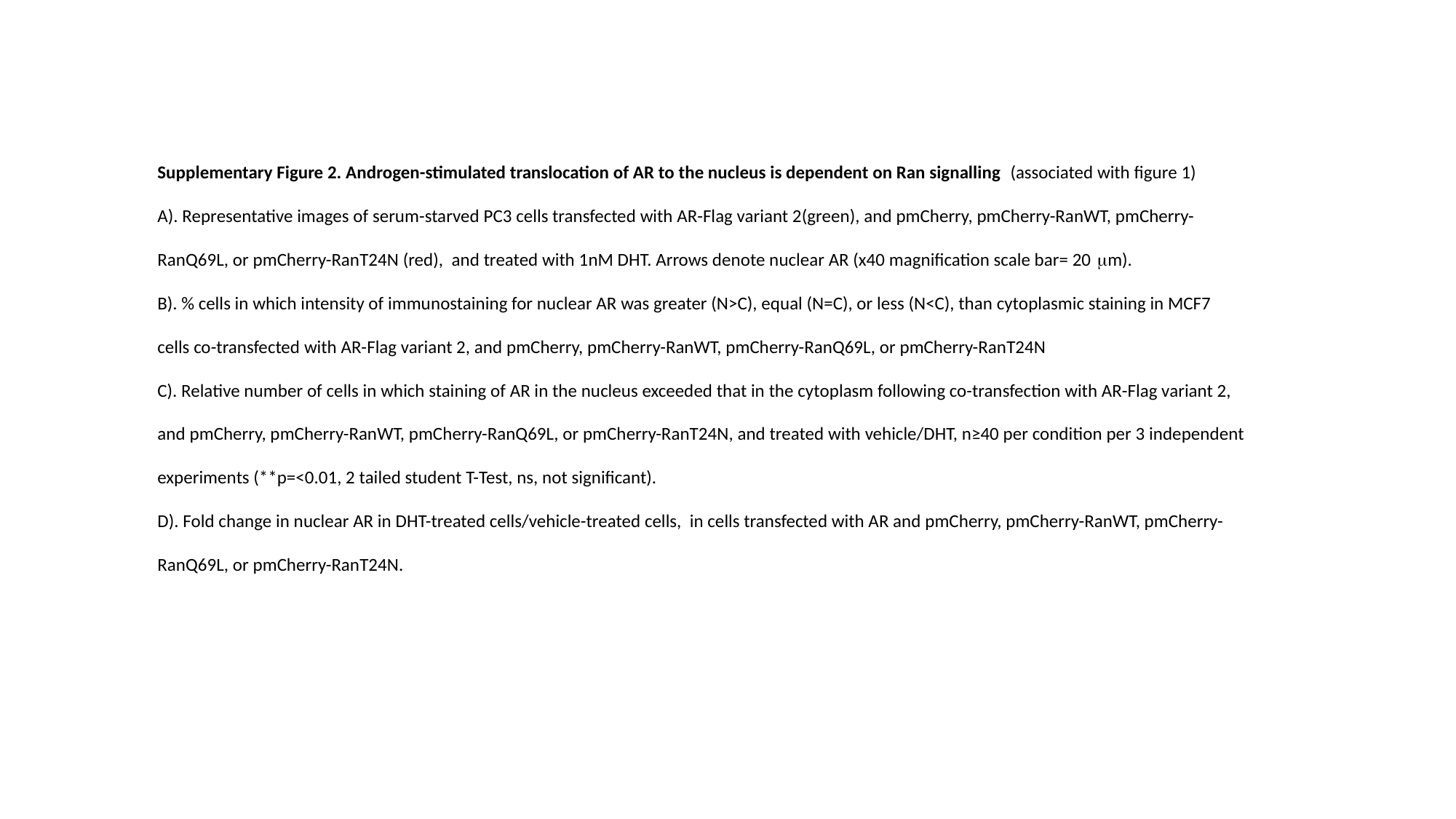

Supplementary Figure 2. Androgen-stimulated translocation of AR to the nucleus is dependent on Ran signalling (associated with figure 1)
A). Representative images of serum-starved PC3 cells transfected with AR-Flag variant 2(green), and pmCherry, pmCherry-RanWT, pmCherry-RanQ69L, or pmCherry-RanT24N (red), and treated with 1nM DHT. Arrows denote nuclear AR (x40 magnification scale bar= 20 mm).
B). % cells in which intensity of immunostaining for nuclear AR was greater (N>C), equal (N=C), or less (N<C), than cytoplasmic staining in MCF7 cells co-transfected with AR-Flag variant 2, and pmCherry, pmCherry-RanWT, pmCherry-RanQ69L, or pmCherry-RanT24N
C). Relative number of cells in which staining of AR in the nucleus exceeded that in the cytoplasm following co-transfection with AR-Flag variant 2, and pmCherry, pmCherry-RanWT, pmCherry-RanQ69L, or pmCherry-RanT24N, and treated with vehicle/DHT, n≥40 per condition per 3 independent experiments (**p=<0.01, 2 tailed student T-Test, ns, not significant).
D). Fold change in nuclear AR in DHT-treated cells/vehicle-treated cells, in cells transfected with AR and pmCherry, pmCherry-RanWT, pmCherry-RanQ69L, or pmCherry-RanT24N.
