# Supplementary material for: B-type Plexins promote the GTPase activity of Ran to affect androgen receptor nuclear translocation in prostate cancer
Source: Cancer Gene Ther. 2023 Aug 10;30(11):1513–23. doi: 10.1038/s41417-023-00655-6 (PMC10645588; doi:10.1038/s41417-023-00655-6)
Supplement: Supplementary file 4 — Supplementary Figure 3 [file 41417_2023_655_MOESM4_ESM.pptx]

## Slide 1
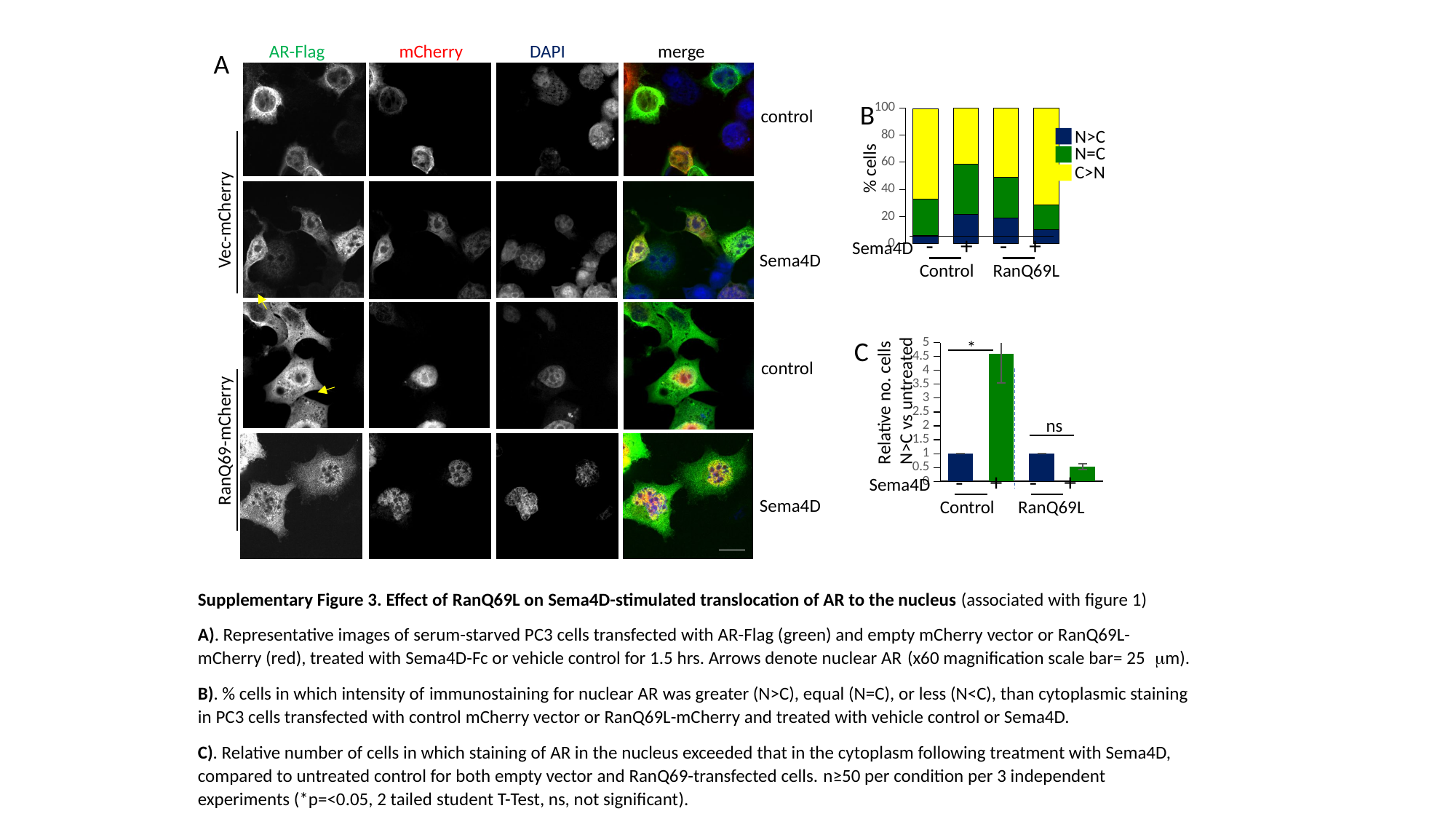

AR-Flag
mCherry
DAPI
merge
A
control
Vec-mCherry
control
RanQ69-mCherry
Sema4D
Sema4D
B
### Chart
| Category | N>C | C=N | C>N |
|---|---|---|---|
| VEC PBS | 5.760000000000001 | 27.099999999999998 | 66.66666666666667 |
| VEC S4D | 21.5 | 37.26666666666667 | 41.06666666666666 |
| Q69L PBS | 18.73333333333333 | 30.03333333333333 | 51.03333333333333 |
| Q69L S4D | 10.346666666666666 | 18.316666666666666 | 71.05 |N>C
N=C
C>N
% cells
Sema4D - + - +
Control
RanQ69L
C
*
### Chart
| Category | |
|---|---|Relative no. cells N>C vs untreated
ns
Sema4D - + - +
Control
RanQ69L
Supplementary Figure 3. Effect of RanQ69L on Sema4D-stimulated translocation of AR to the nucleus (associated with figure 1)
A). Representative images of serum-starved PC3 cells transfected with AR-Flag (green) and empty mCherry vector or RanQ69L- mCherry (red), treated with Sema4D-Fc or vehicle control for 1.5 hrs. Arrows denote nuclear AR (x60 magnification scale bar= 25 mm).
B). % cells in which intensity of immunostaining for nuclear AR was greater (N>C), equal (N=C), or less (N<C), than cytoplasmic staining in PC3 cells transfected with control mCherry vector or RanQ69L-mCherry and treated with vehicle control or Sema4D.
C). Relative number of cells in which staining of AR in the nucleus exceeded that in the cytoplasm following treatment with Sema4D, compared to untreated control for both empty vector and RanQ69-transfected cells. n≥50 per condition per 3 independent experiments (*p=<0.05, 2 tailed student T-Test, ns, not significant).
