# Supplementary material for: B-type Plexins promote the GTPase activity of Ran to affect androgen receptor nuclear translocation in prostate cancer
Source: Cancer Gene Ther. 2023 Aug 10;30(11):1513–23. doi: 10.1038/s41417-023-00655-6 (PMC10645588; doi:10.1038/s41417-023-00655-6)
Supplement: Supplementary file 5 — Supplementary Figure 4 [file 41417_2023_655_MOESM5_ESM.pptx]

## Slide 1
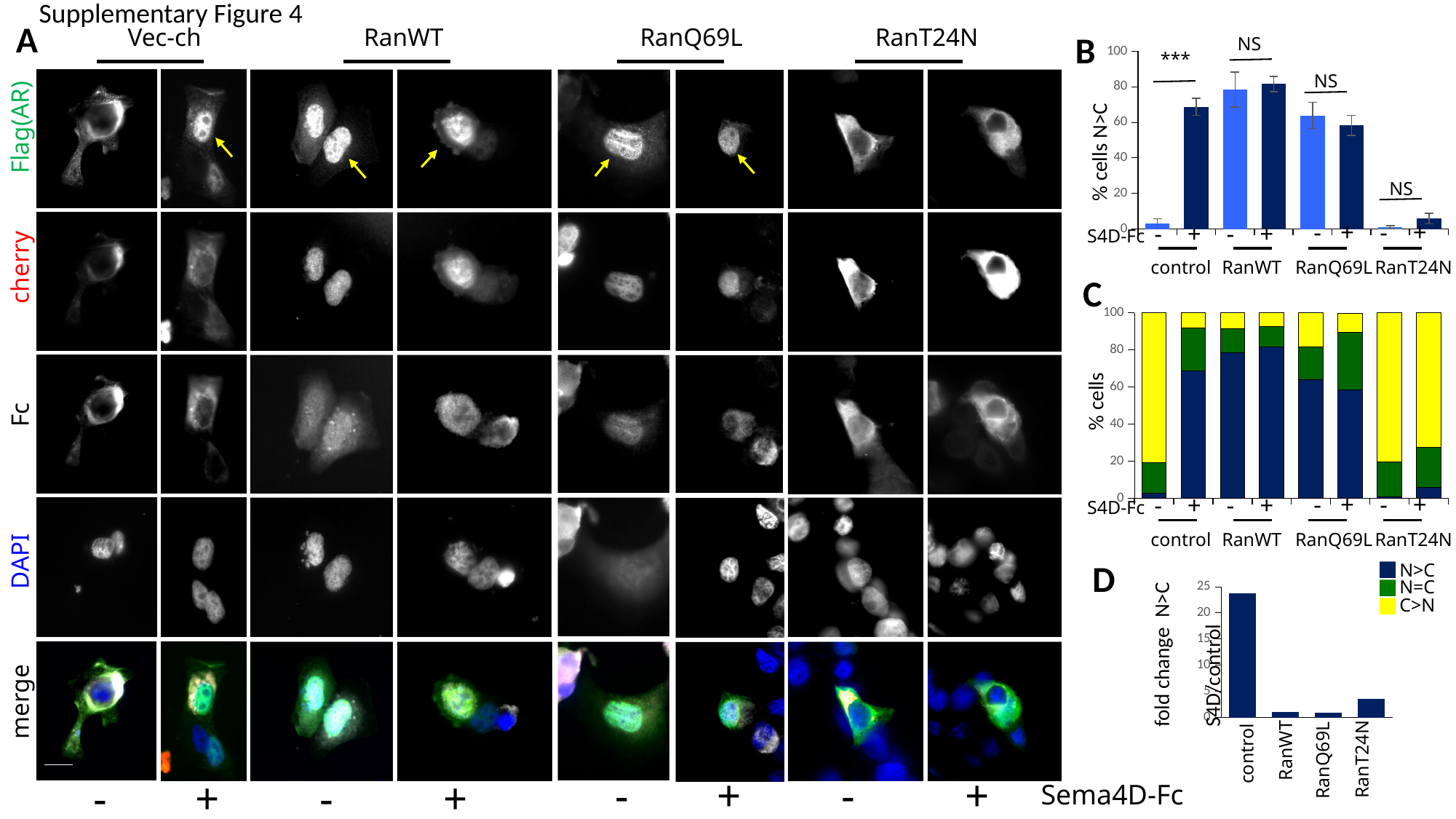

Supplementary Figure 4
A
Vec-ch
RanWT
RanQ69L
RanT24N
B
NS
### Chart
| Category | |
|---|---|***
NS
% cells N>C
NS
- + - +
S4D-Fc - + - +
control
RanWT
RanQ69L
RanT24N
Flag(AR)
cherry
C
### Chart
| Category | | | |
|---|---|---|---|- + - +
S4D-Fc - + - +
control
RanWT
RanQ69L
RanT24N
% cells
Fc
DAPI
D
N>C
N=C
C>N
### Chart
| Category | |
|---|---|fold change N>C
S4D/control
RanWT
control
RanT24N
RanQ69L
merge
- + - +
- + - +
Sema4D-Fc

## Slide 2
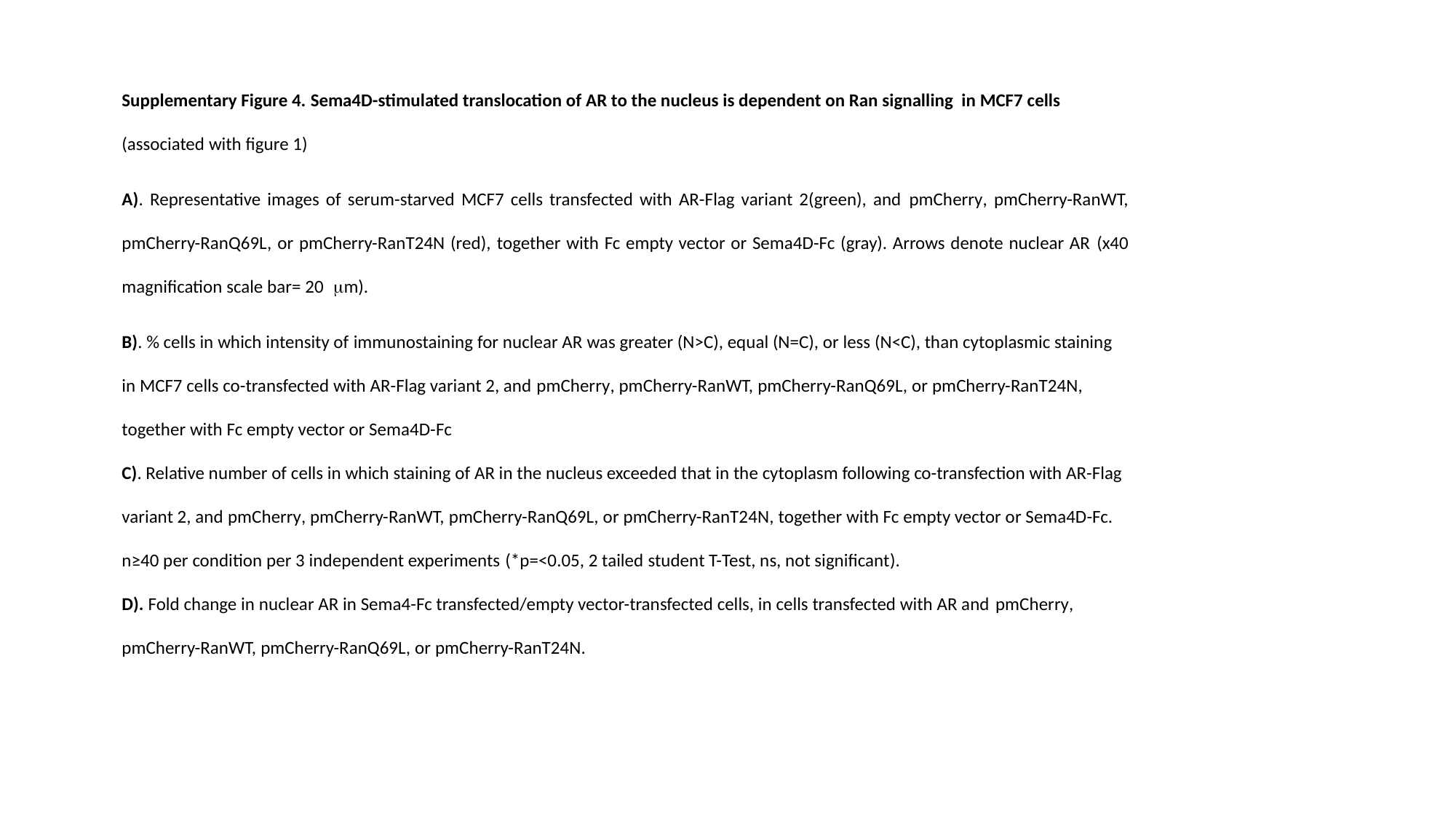

Supplementary Figure 4. Sema4D-stimulated translocation of AR to the nucleus is dependent on Ran signalling in MCF7 cells (associated with figure 1)
A). Representative images of serum-starved MCF7 cells transfected with AR-Flag variant 2(green), and pmCherry, pmCherry-RanWT, pmCherry-RanQ69L, or pmCherry-RanT24N (red), together with Fc empty vector or Sema4D-Fc (gray). Arrows denote nuclear AR (x40 magnification scale bar= 20 mm).
B). % cells in which intensity of immunostaining for nuclear AR was greater (N>C), equal (N=C), or less (N<C), than cytoplasmic staining in MCF7 cells co-transfected with AR-Flag variant 2, and pmCherry, pmCherry-RanWT, pmCherry-RanQ69L, or pmCherry-RanT24N, together with Fc empty vector or Sema4D-Fc
C). Relative number of cells in which staining of AR in the nucleus exceeded that in the cytoplasm following co-transfection with AR-Flag variant 2, and pmCherry, pmCherry-RanWT, pmCherry-RanQ69L, or pmCherry-RanT24N, together with Fc empty vector or Sema4D-Fc. n≥40 per condition per 3 independent experiments (*p=<0.05, 2 tailed student T-Test, ns, not significant).
D). Fold change in nuclear AR in Sema4-Fc transfected/empty vector-transfected cells, in cells transfected with AR and pmCherry, pmCherry-RanWT, pmCherry-RanQ69L, or pmCherry-RanT24N.
