# Supplementary material for: B-type Plexins promote the GTPase activity of Ran to affect androgen receptor nuclear translocation in prostate cancer
Source: Cancer Gene Ther. 2023 Aug 10;30(11):1513–23. doi: 10.1038/s41417-023-00655-6 (PMC10645588; doi:10.1038/s41417-023-00655-6)
Supplement: Supplementary file 6 — Supplementary Figure 5 [file 41417_2023_655_MOESM6_ESM.pptx]

## Slide 1
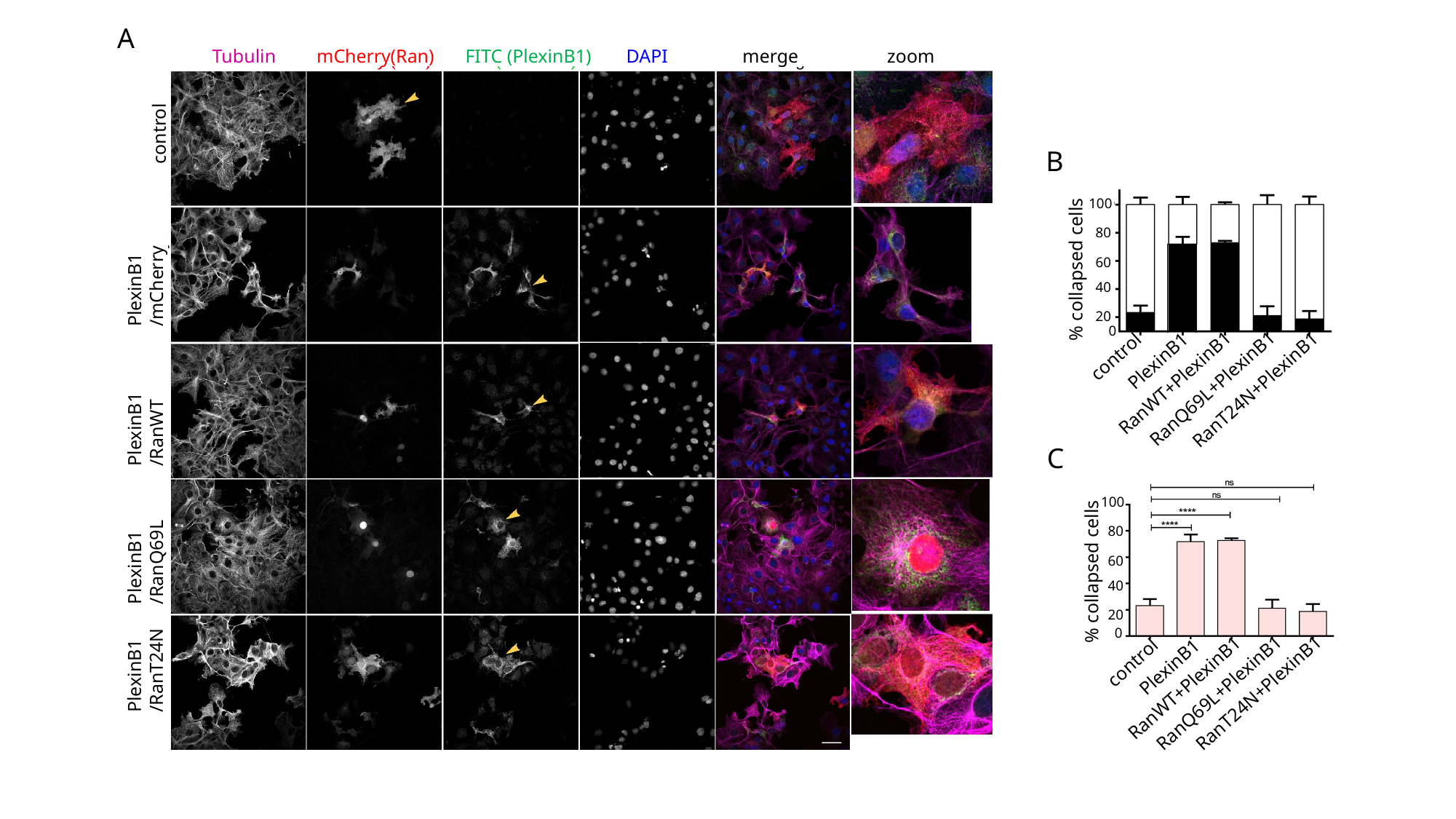

A
Tubulin
mCherry(Ran)
FITC (PlexinB1)
DAPI
merge
zoom
control
PlexinB1
/mCherry
PlexinB1
/RanWT
PlexinB1
/RanQ69L
PlexinB1
/RanT24N
B
D
% collapsed cells
control
PlexinB1
RanWT+PlexinB1
RanQ69L+PlexinB1
RanT24N+PlexinB1
100
80
60
40
20
0
C
control
100
80
60
40
20
0
% collapsed cells
control
PlexinB1
RanWT+PlexinB1
RanQ69L+PlexinB1
RanT24N+PlexinB1
Sema4D

## Slide 2
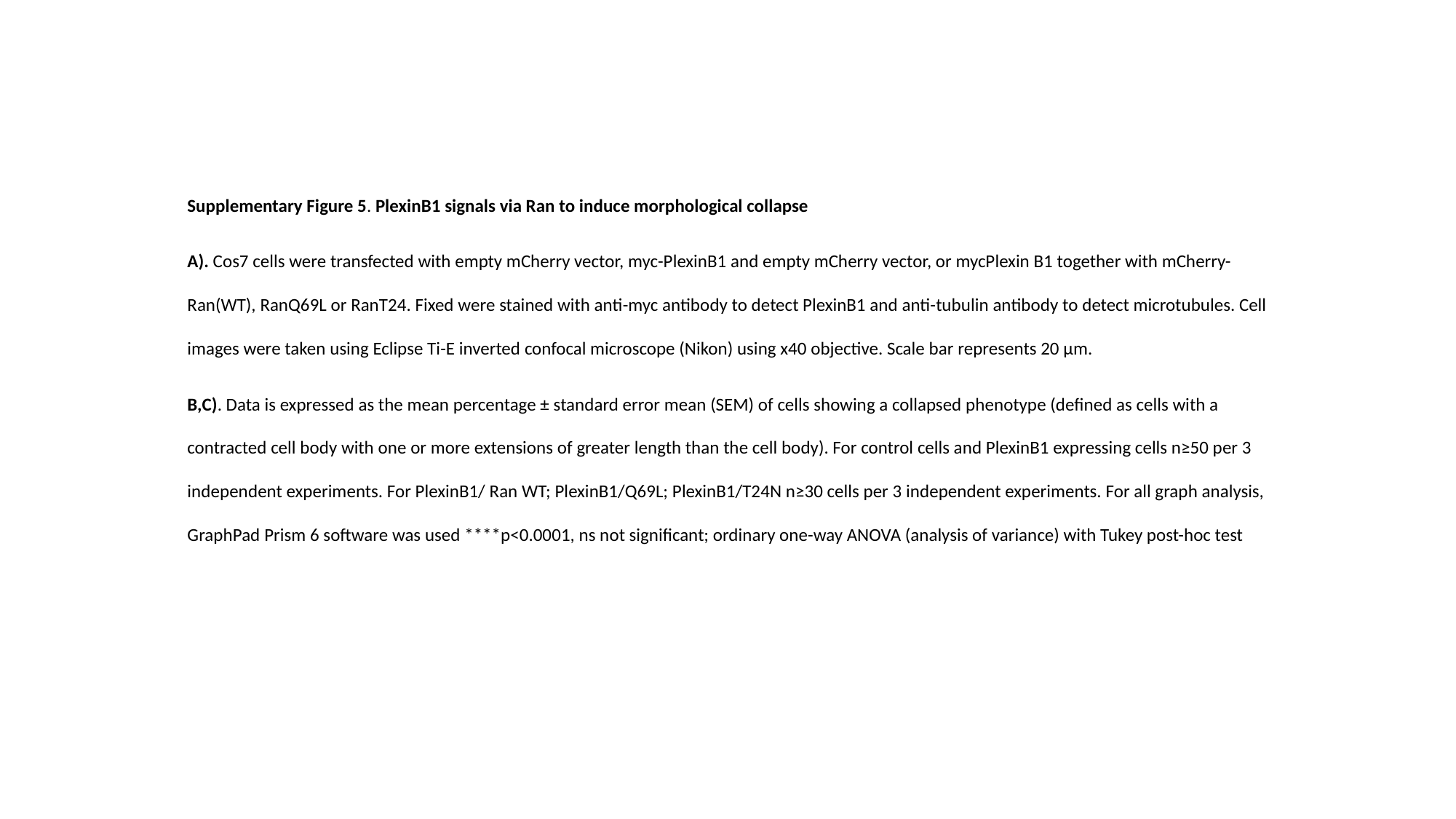

Supplementary Figure 5. PlexinB1 signals via Ran to induce morphological collapse
A). Cos7 cells were transfected with empty mCherry vector, myc-PlexinB1 and empty mCherry vector, or mycPlexin B1 together with mCherry-Ran(WT), RanQ69L or RanT24. Fixed were stained with anti-myc antibody to detect PlexinB1 and anti-tubulin antibody to detect microtubules. Cell images were taken using Eclipse Ti-E inverted confocal microscope (Nikon) using x40 objective. Scale bar represents 20 µm.
B,C). Data is expressed as the mean percentage ± standard error mean (SEM) of cells showing a collapsed phenotype (defined as cells with a contracted cell body with one or more extensions of greater length than the cell body). For control cells and PlexinB1 expressing cells n≥50 per 3 independent experiments. For PlexinB1/ Ran WT; PlexinB1/Q69L; PlexinB1/T24N n≥30 cells per 3 independent experiments. For all graph analysis, GraphPad Prism 6 software was used ****p<0.0001, ns not significant; ordinary one-way ANOVA (analysis of variance) with Tukey post-hoc test
