# Supplementary material for: B-type Plexins promote the GTPase activity of Ran to affect androgen receptor nuclear translocation in prostate cancer
Source: Cancer Gene Ther. 2023 Aug 10;30(11):1513–23. doi: 10.1038/s41417-023-00655-6 (PMC10645588; doi:10.1038/s41417-023-00655-6)
Supplement: Supplementary file 7 — Supplementary Figure 6 [file 41417_2023_655_MOESM7_ESM.pptx]

## Slide 1
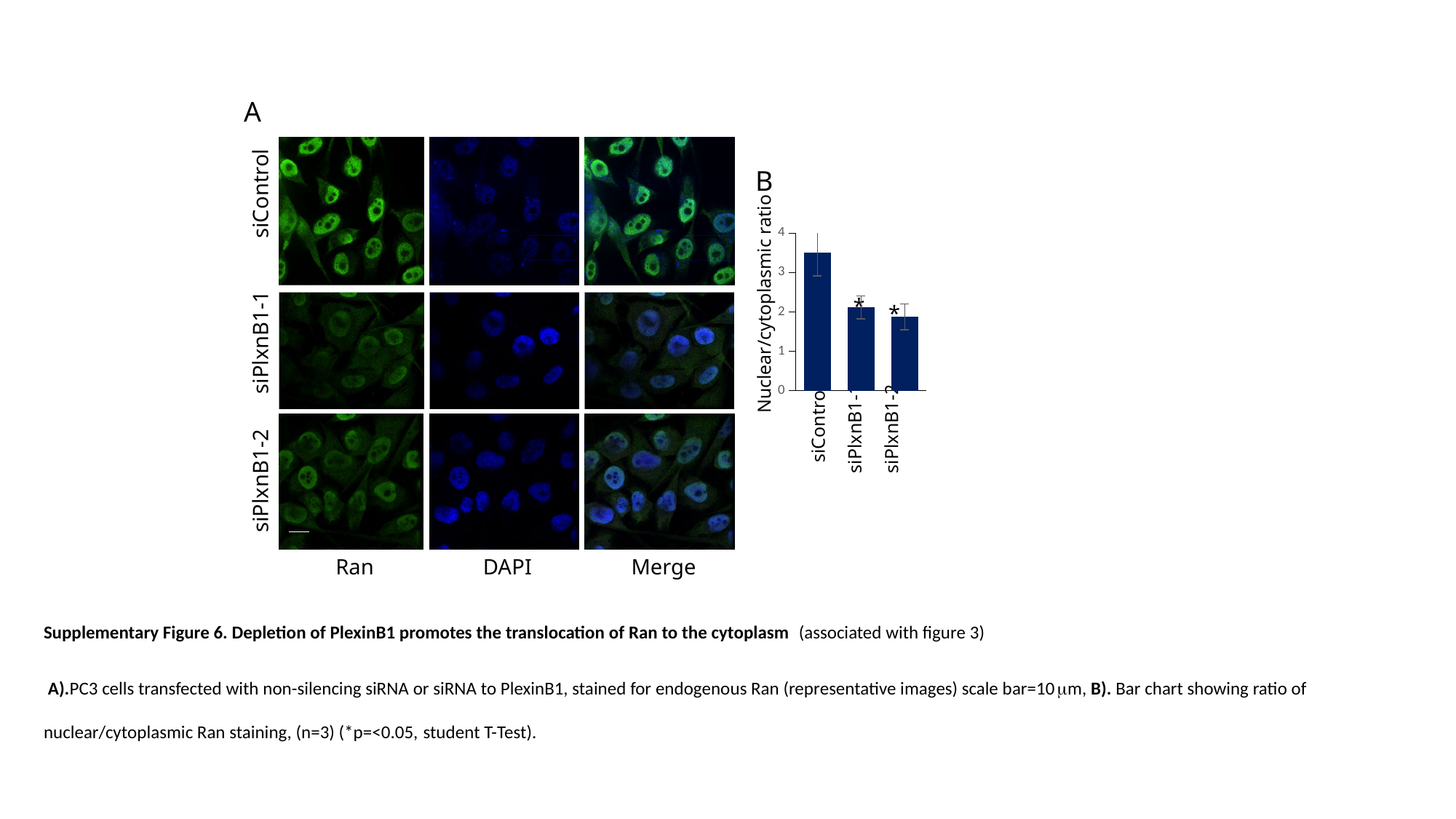

A
siControl
siPlxnB1-1
siPlxnB1-2
Ran
DAPI
Merge
B
Nuclear/cytoplasmic ratio
siControl
siPlxnB1-1
siPlxnB1-2
### Chart
| Category | |
|---|---|*
*
Supplementary Figure 6. Depletion of PlexinB1 promotes the translocation of Ran to the cytoplasm (associated with figure 3)
 A).PC3 cells transfected with non-silencing siRNA or siRNA to PlexinB1, stained for endogenous Ran (representative images) scale bar=10mm, B). Bar chart showing ratio of nuclear/cytoplasmic Ran staining, (n=3) (*p=<0.05, student T-Test).
