# Supplementary material for: B-type Plexins promote the GTPase activity of Ran to affect androgen receptor nuclear translocation in prostate cancer
Source: Cancer Gene Ther. 2023 Aug 10;30(11):1513–23. doi: 10.1038/s41417-023-00655-6 (PMC10645588; doi:10.1038/s41417-023-00655-6)
Supplement: Supplementary file 8 — Supplementary Figure 7 [file 41417_2023_655_MOESM8_ESM.pptx]

## Slide 1
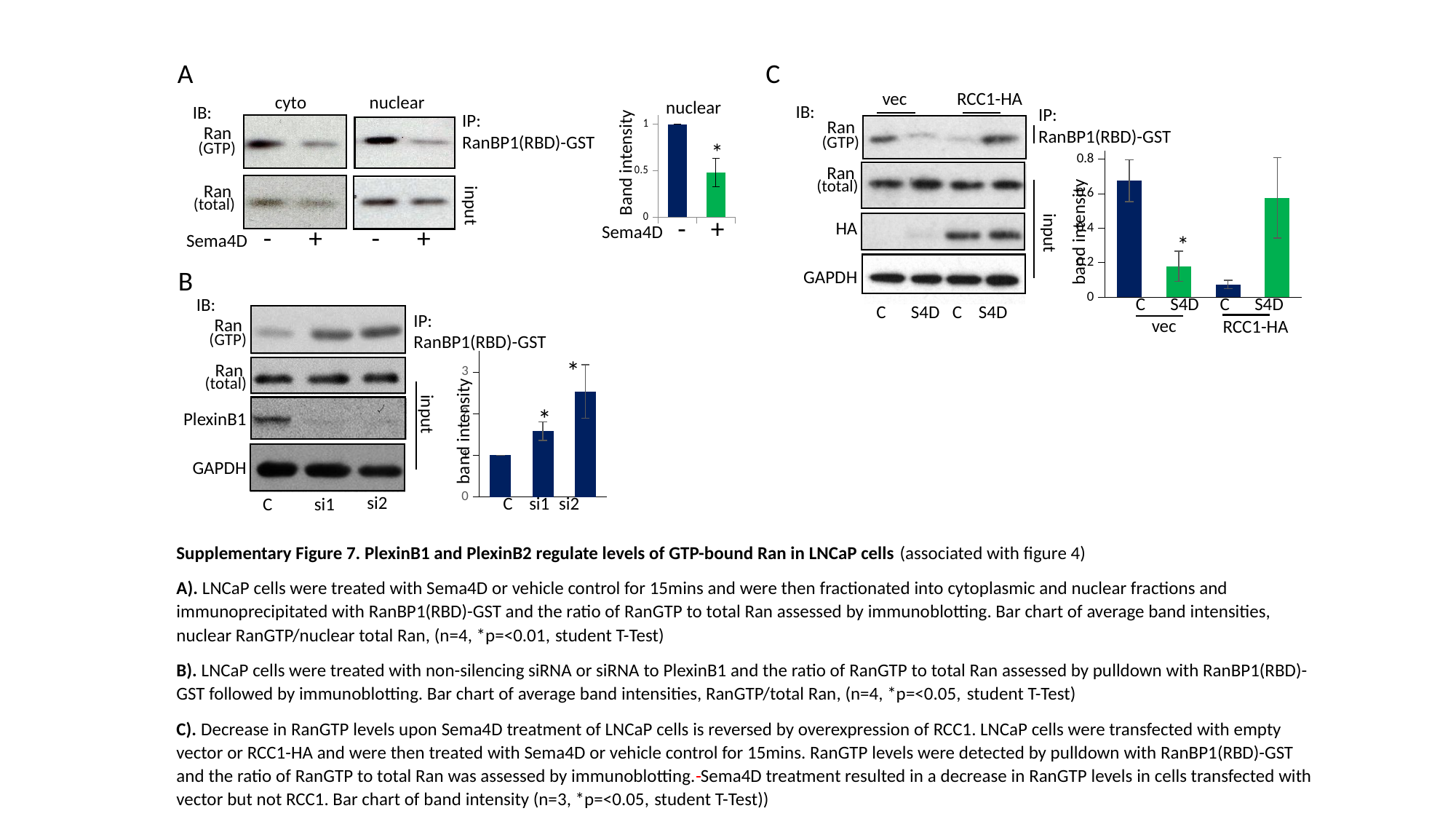

A
C
vec
RCC1-HA
IB:
 IP:
 RanBP1(RBD)-GST
Ran
(GTP)
### Chart
| Category | |
|---|---|C S4D C S4D
vec
RCC1-HA
band intensity
 Ran
(total)
input
HA
GAPDH
C S4D C S4D
cyto
nuclear
IB:
 IP:
 RanBP1(RBD)-GST
Ran
(GTP)
 Ran
(total)
input
 - + - +
Sema4D
nuclear
### Chart
| Category | |
|---|---|
*
Band intensity
Sema4D - +
*
B
IB:
 IP:
 RanBP1(RBD)-GST
Ran
(GTP)
### Chart
| Category | |
|---|---|*
*
band intensity
 Ran
(total)
input
PlexinB1
GAPDH
si2
C
si1
si2
C
si1
Supplementary Figure 7. PlexinB1 and PlexinB2 regulate levels of GTP-bound Ran in LNCaP cells (associated with figure 4)
A). LNCaP cells were treated with Sema4D or vehicle control for 15mins and were then fractionated into cytoplasmic and nuclear fractions and immunoprecipitated with RanBP1(RBD)-GST and the ratio of RanGTP to total Ran assessed by immunoblotting. Bar chart of average band intensities, nuclear RanGTP/nuclear total Ran, (n=4, *p=<0.01, student T-Test)
B). LNCaP cells were treated with non-silencing siRNA or siRNA to PlexinB1 and the ratio of RanGTP to total Ran assessed by pulldown with RanBP1(RBD)-GST followed by immunoblotting. Bar chart of average band intensities, RanGTP/total Ran, (n=4, *p=<0.05, student T-Test)
C). Decrease in RanGTP levels upon Sema4D treatment of LNCaP cells is reversed by overexpression of RCC1. LNCaP cells were transfected with empty vector or RCC1-HA and were then treated with Sema4D or vehicle control for 15mins. RanGTP levels were detected by pulldown with RanBP1(RBD)-GST and the ratio of RanGTP to total Ran was assessed by immunoblotting. Sema4D treatment resulted in a decrease in RanGTP levels in cells transfected with vector but not RCC1. Bar chart of band intensity (n=3, *p=<0.05, student T-Test))
