# Supplementary material for: B-type Plexins promote the GTPase activity of Ran to affect androgen receptor nuclear translocation in prostate cancer
Source: Cancer Gene Ther. 2023 Aug 10;30(11):1513–23. doi: 10.1038/s41417-023-00655-6 (PMC10645588; doi:10.1038/s41417-023-00655-6)
Supplement: Supplementary file 9 — Supplementary Figure 8 [file 41417_2023_655_MOESM9_ESM.pptx]

## Slide 1
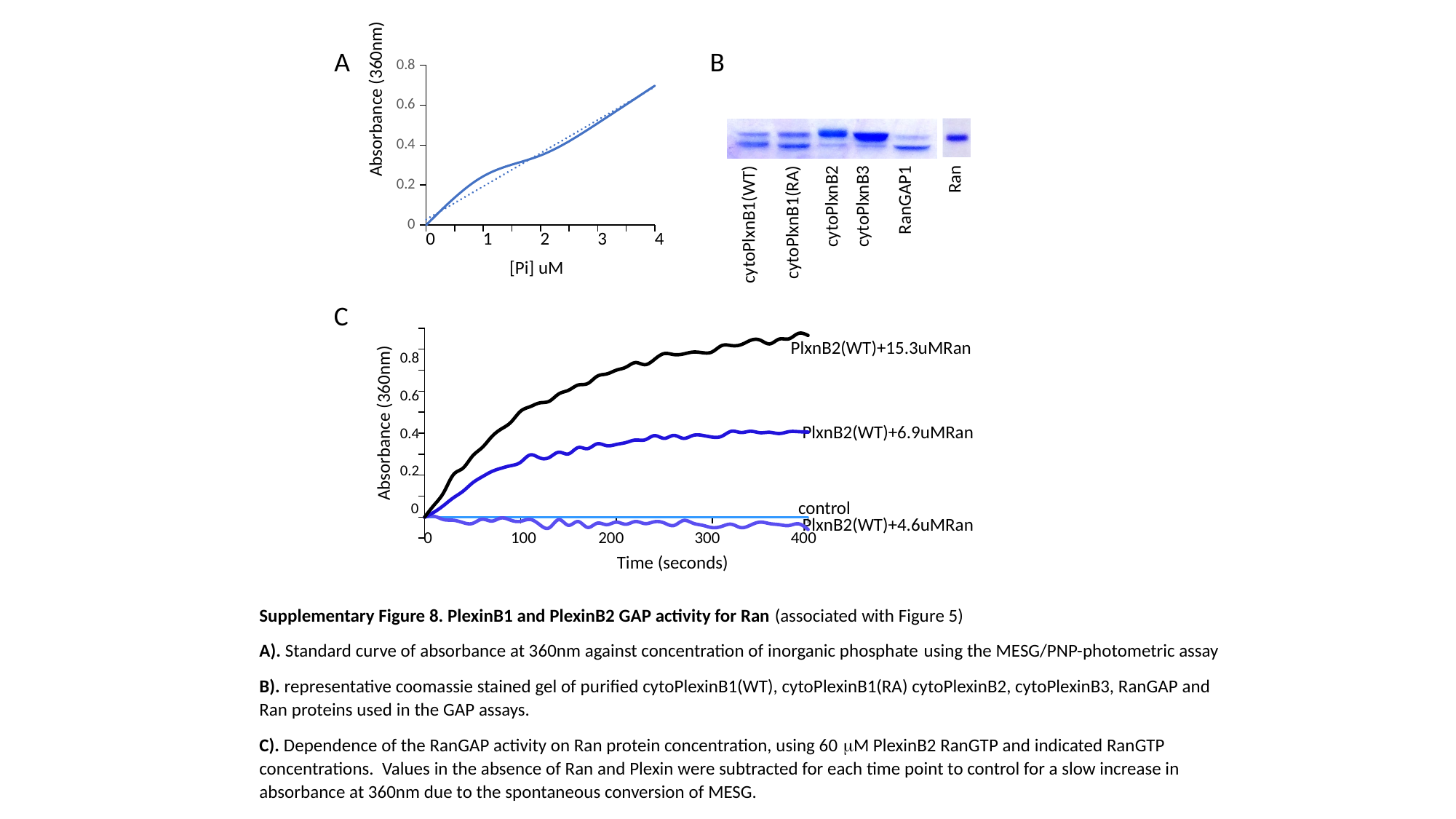

### Chart
| Category | |
|---|---|A
B
Ran
RanGAP1
cytoPlxnB2
cytoPlxnB3
cytoPlxnB1(RA)
cytoPlxnB1(WT)
0
1
2
3
4
[Pi] uM
C
### Chart
| Category | B2 1/15 RAN | B2 1/33 RAN | B2 1/50 RAN | B2 O RAN |
|---|---|---|---|---|PlxnB2(WT)+15.3uMRan
0.8
0.6
0.4
0.2
0
Absorbance (360nm)
PlxnB2(WT)+6.9uMRan
control
PlxnB2(WT)+4.6uMRan
0
100
200
300
400
Time (seconds)
Absorbance (360nm)
Supplementary Figure 8. PlexinB1 and PlexinB2 GAP activity for Ran (associated with Figure 5)
A). Standard curve of absorbance at 360nm against concentration of inorganic phosphate using the MESG/PNP-photometric assay
B). representative coomassie stained gel of purified cytoPlexinB1(WT), cytoPlexinB1(RA) cytoPlexinB2, cytoPlexinB3, RanGAP and Ran proteins used in the GAP assays.
C). Dependence of the RanGAP activity on Ran protein concentration, using 60 mM PlexinB2 RanGTP and indicated RanGTP concentrations. Values in the absence of Ran and Plexin were subtracted for each time point to control for a slow increase in absorbance at 360nm due to the spontaneous conversion of MESG.
