# Supplementary material for: B-type Plexins promote the GTPase activity of Ran to affect androgen receptor nuclear translocation in prostate cancer
Source: Cancer Gene Ther. 2023 Aug 10;30(11):1513–23. doi: 10.1038/s41417-023-00655-6 (PMC10645588; doi:10.1038/s41417-023-00655-6)
Supplement: Supplementary file 10 — Supplementary Figure 9 [file 41417_2023_655_MOESM10_ESM.pptx]

## Slide 1
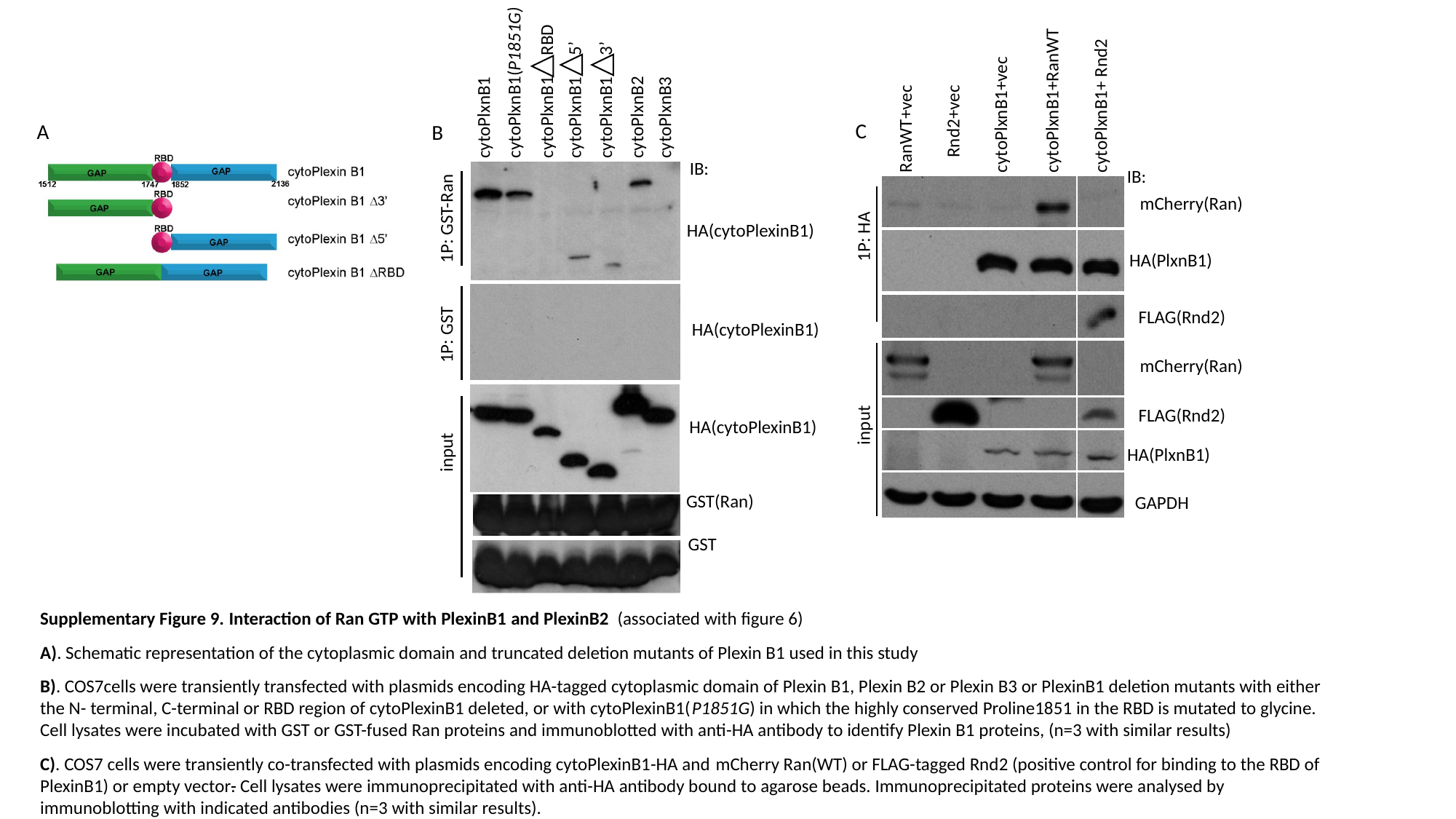

cytoPlxnB1+RanWT
cytoPlxnB1+ Rnd2
cytoPlxnB1+vec
RanWT+vec
Rnd2+vec
IB:
mCherry(Ran)
1P: HA
HA(PlxnB1)
FLAG(Rnd2)
mCherry(Ran)
FLAG(Rnd2)
input
HA(PlxnB1)
GAPDH
cytoPlxnB1 RBD
cytoPlxnB1 5’
cytoPlxnB1 3’
cytoPlxnB1(P1851G)
cytoPlxnB1
cytoPlxnB2
cytoPlxnB3
IB:
1P: GST-Ran
HA(cytoPlexinB1)
HA(cytoPlexinB1)
1P: GST
HA(cytoPlexinB1)
input
GST(Ran)
GST
C
A
B
Supplementary Figure 9. Interaction of Ran GTP with PlexinB1 and PlexinB2 (associated with figure 6)
A). Schematic representation of the cytoplasmic domain and truncated deletion mutants of Plexin B1 used in this study
B). COS7cells were transiently transfected with plasmids encoding HA-tagged cytoplasmic domain of Plexin B1, Plexin B2 or Plexin B3 or PlexinB1 deletion mutants with either the N- terminal, C-terminal or RBD region of cytoPlexinB1 deleted, or with cytoPlexinB1(P1851G) in which the highly conserved Proline1851 in the RBD is mutated to glycine. Cell lysates were incubated with GST or GST-fused Ran proteins and immunoblotted with anti-HA antibody to identify Plexin B1 proteins, (n=3 with similar results)
C). COS7 cells were transiently co-transfected with plasmids encoding cytoPlexinB1-HA and mCherry Ran(WT) or FLAG-tagged Rnd2 (positive control for binding to the RBD of PlexinB1) or empty vector. Cell lysates were immunoprecipitated with anti-HA antibody bound to agarose beads. Immunoprecipitated proteins were analysed by immunoblotting with indicated antibodies (n=3 with similar results).
