# Supplementary material for: B-type Plexins promote the GTPase activity of Ran to affect androgen receptor nuclear translocation in prostate cancer
Source: Cancer Gene Ther. 2023 Aug 10;30(11):1513–23. doi: 10.1038/s41417-023-00655-6 (PMC10645588; doi:10.1038/s41417-023-00655-6)
Supplement: Supplementary file 11 — Supplementary Figure 10 [file 41417_2023_655_MOESM11_ESM.pptx]

## Slide 1
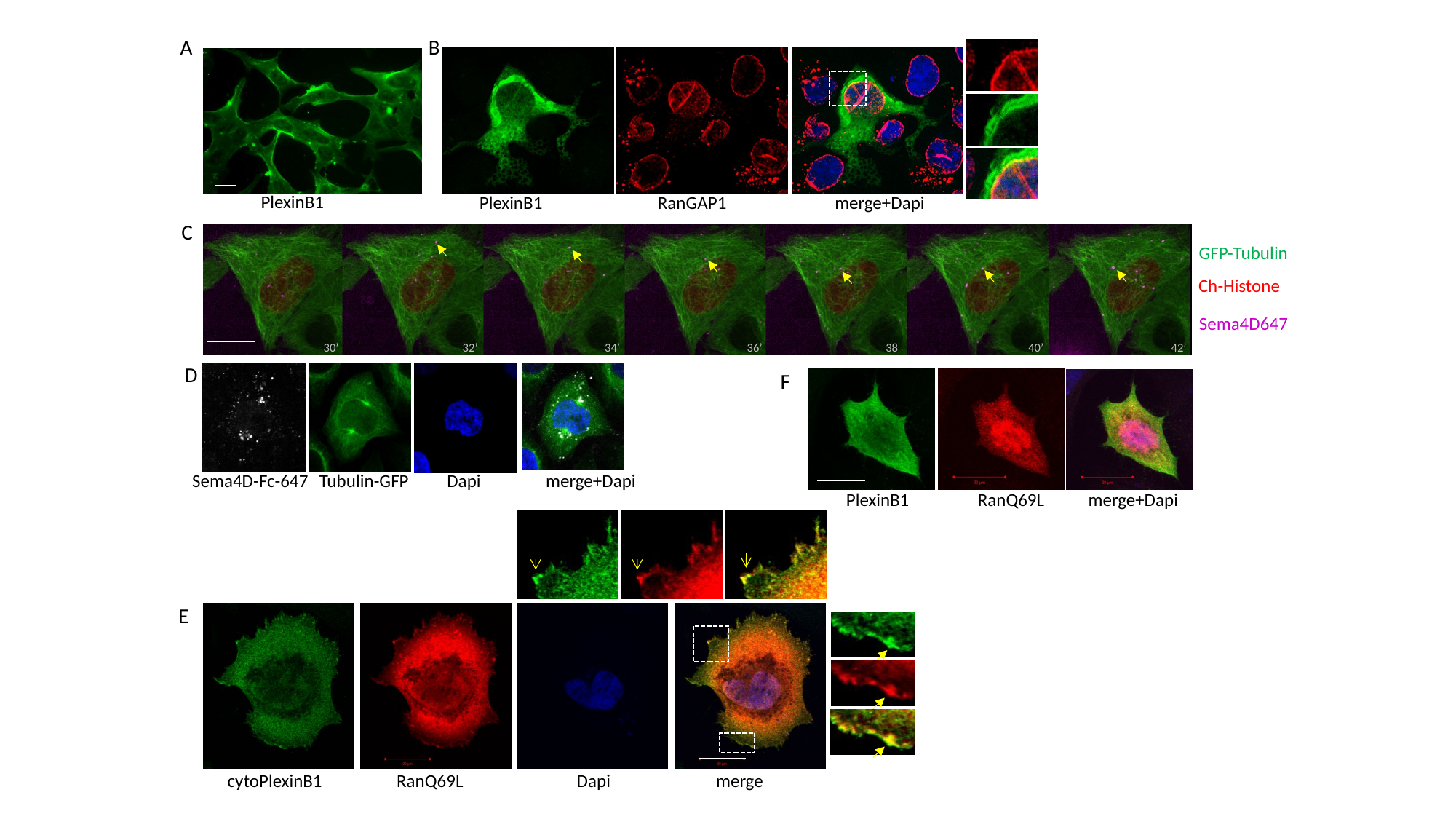

A
B
PlexinB1
RanGAP1
merge+Dapi
PlexinB1
C
E
GFP-Tubulin
Ch-Histone
Sema4D647
30’
32’
34’
36’
38
40’
42’
S4D647
D
Sema4D-Fc-647
Tubulin-GFP
Dapi
merge+Dapi
F
PlexinB1
RanQ69L
merge+Dapi
cytoPlexinB1
RanQ69L
Dapi
merge
E

## Slide 2
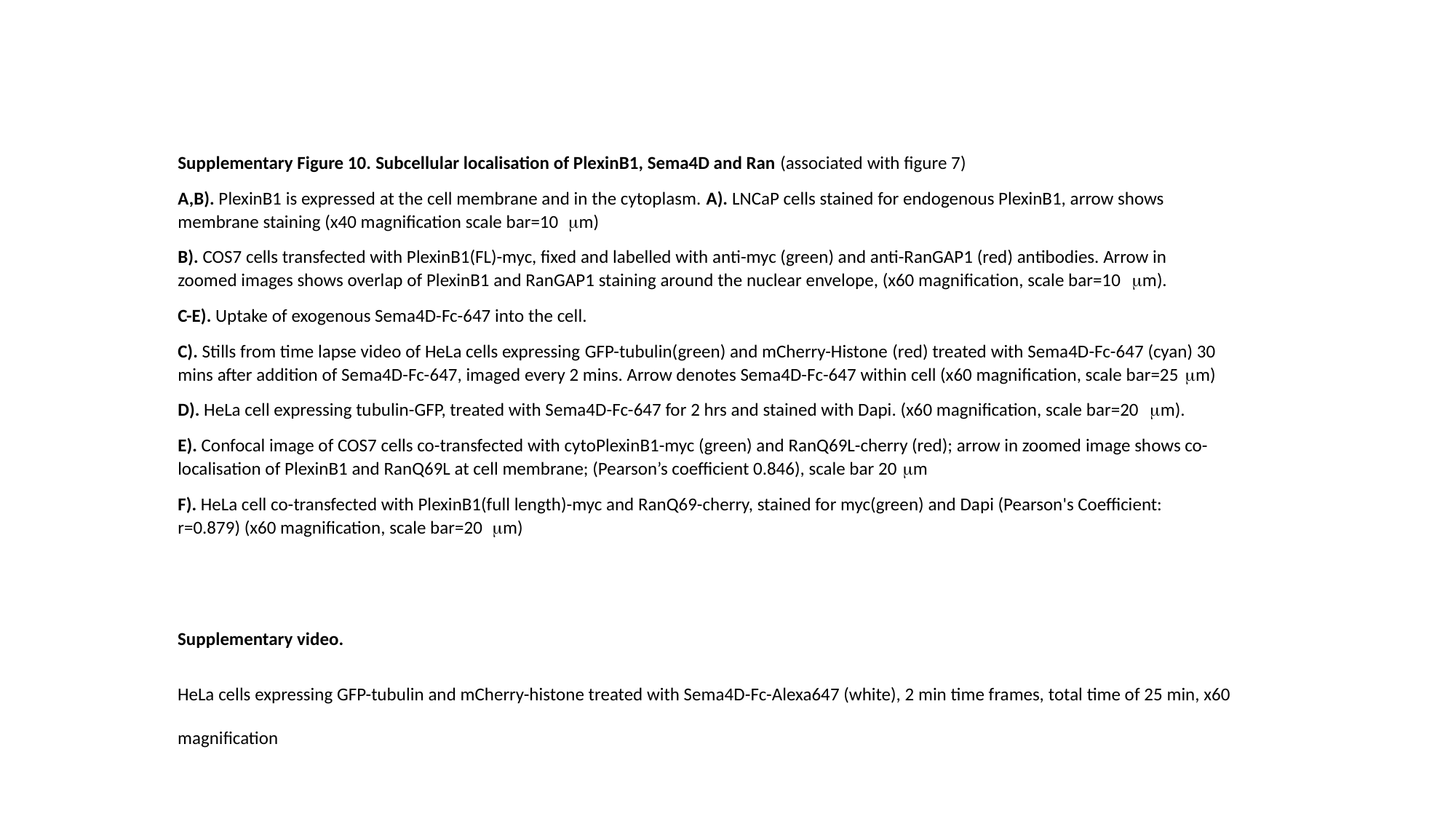

Supplementary Figure 10. Subcellular localisation of PlexinB1, Sema4D and Ran (associated with figure 7)
A,B). PlexinB1 is expressed at the cell membrane and in the cytoplasm. A). LNCaP cells stained for endogenous PlexinB1, arrow shows membrane staining (x40 magnification scale bar=10 mm)
B). COS7 cells transfected with PlexinB1(FL)-myc, fixed and labelled with anti-myc (green) and anti-RanGAP1 (red) antibodies. Arrow in zoomed images shows overlap of PlexinB1 and RanGAP1 staining around the nuclear envelope, (x60 magnification, scale bar=10 mm).
C-E). Uptake of exogenous Sema4D-Fc-647 into the cell.
C). Stills from time lapse video of HeLa cells expressing GFP-tubulin(green) and mCherry-Histone (red) treated with Sema4D-Fc-647 (cyan) 30 mins after addition of Sema4D-Fc-647, imaged every 2 mins. Arrow denotes Sema4D-Fc-647 within cell (x60 magnification, scale bar=25 mm)
D). HeLa cell expressing tubulin-GFP, treated with Sema4D-Fc-647 for 2 hrs and stained with Dapi. (x60 magnification, scale bar=20 mm).
E). Confocal image of COS7 cells co-transfected with cytoPlexinB1-myc (green) and RanQ69L-cherry (red); arrow in zoomed image shows co-localisation of PlexinB1 and RanQ69L at cell membrane; (Pearson’s coefficient 0.846), scale bar 20 mm
F). HeLa cell co-transfected with PlexinB1(full length)-myc and RanQ69-cherry, stained for myc(green) and Dapi (Pearson's Coefficient: r=0.879) (x60 magnification, scale bar=20 mm)
Supplementary video.
HeLa cells expressing GFP-tubulin and mCherry-histone treated with Sema4D-Fc-Alexa647 (white), 2 min time frames, total time of 25 min, x60 magnification
