# Supplementary material for: B-type Plexins promote the GTPase activity of Ran to affect androgen receptor nuclear translocation in prostate cancer
Source: Cancer Gene Ther. 2023 Aug 10;30(11):1513–23. doi: 10.1038/s41417-023-00655-6 (PMC10645588; doi:10.1038/s41417-023-00655-6)
Supplement: Supplementary file 12 — Supplementary video [file 41417_2023_655_MOESM12_ESM.docx]

**Supplementary video.** HeLa cells expressing GFP-tubulin and mCherry-histone treated with Sema4D-Fc-Alexa647 (white), 2 min time frames, total time of 25 min, x60 magnification
